# Genome-wide Polygenic Burden of Rare Deleterious Variants in Sudden Unexpected Death in Epilepsy

**DOI:** 10.1016/j.ebiom.2015.07.005

**Published:** 2015-07-10

**Authors:** Costin Leu, Simona Balestrini, Bridget Maher, Laura Hernández-Hernández, Padhraig Gormley, Eija Hämäläinen, Kristin Heggeli, Natasha Schoeler, Jan Novy, Joseph Willis, Vincent Plagnol, Rachael Ellis, Eleanor Reavey, Mary O'Regan, William O. Pickrell, Rhys H. Thomas, Seo-Kyung Chung, Norman Delanty, Jacinta M. McMahon, Stephen Malone, Lynette G. Sadleir, Samuel F. Berkovic, Lina Nashef, Sameer M. Zuberi, Mark I. Rees, Gianpiero L. Cavalleri, Josemir W. Sander, Elaine Hughes, J. Helen Cross, Ingrid E. Scheffer, Aarno Palotie, Sanjay M. Sisodiya

**Affiliations:** a NIHR University College London Hospitals Biomedical Research Centre, Department of Clinical and Experimental Epilepsy, UCL Institute of Neurology, London, UK; b The Epilepsy Society, Chalfont-St-Peter, Bucks, UK; c Neuroscience Department, Polytechnic University of Marche, Ancona, Italy; d Analytic and Translational Genetics Unit, Department of Medicine, Massachusetts General Hospital, Boston, MA, USA; e Program in Medical and Population Genetics, The Broad Institute of MIT and Harvard, Cambridge, MA, USA; f The Stanley Center for Psychiatric Research, The Broad Institute of MIT and Harvard, Cambridge, MA, USA; g Psychiatric & Neurodevelopmental Genetics Unit, Department of Psychiatry, Massachusetts General Hospital, Boston, MA, USA; h Institute for Molecular Medicine Finland, University of Helsinki, Helsinki, Finland; i Department of Clinical Neurosciences, Centre Hospitalier Universitaire Vaudois (CHUV) and University of Lausanne, Lausanne, Switzerland; j University College London Genetics Institute, London, UK; k Paediatric Neurosciences Research Group, Royal Hospital for Sick Children, Glasgow, UK; l West of Scotland Genetic Services, Southern General Hospital, Glasgow, UK; m Wales Epilepsy Research Network, Institute of Life Science, College of Medicine, Swansea University, Swansea, UK; n Department of Neurology, Beaumont Hospital, Dublin, Ireland; o Departments of Medicine and Neurology, University of Melbourne, Austin Health, Melbourne, Australia; p Department of Neurosciences, Lady Cilento Children's Hospital, Brisbane, Queensland, Australia; q Department of Paediatrics, School of Medicine and Health Sciences, University of Otago, Wellington, New Zealand; r Department of Neurology, King's College Hospital, London, UK; s School of Medicine, University of Glasgow, Glasgow, UK; t Molecular and Cellular Therapeutics Department, Royal College of Surgeons in Ireland, Dublin, Ireland; u Children's Neurosciences, Evelina Children's Hospital at Guys and St Thomas' NHS Foundation Trust, Kings Health Partners Academic Health Science Centre, London, UK; v UCL Institute of Child Health, Great Ormond Street Hospital for Children NHS Foundation Trust, London, UK; w Young Epilepsy, Lingfield, UK; x Department of Paediatrics, University of Melbourne, Royal Children's Hospital, Parkville, Australia; y Florey Institute of Neuroscience and Mental Health, Melbourne, Australia; z Department of Neurology, Massachusetts General Hospital, Boston, MA, USA

**Keywords:** AED, anti-epileptic drug, MAF, minor allele frequency, *n*, number, QC, quality control, SUDEP, sudden unexpected death in epilepsy, WES, whole-exome sequencing, Epilepsy, Death, Mortality, Severity, Association, Burden

## Abstract

Sudden unexpected death in epilepsy (SUDEP) represents the most severe degree of the spectrum of epilepsy severity and is the commonest cause of epilepsy-related premature mortality. The precise pathophysiology and the genetic architecture of SUDEP remain elusive. Aiming to elucidate the genetic basis of SUDEP, we analysed rare, protein-changing variants from whole-exome sequences of 18 people who died of SUDEP, 87 living people with epilepsy and 1479 non-epilepsy disease controls. Association analysis revealed a significantly increased genome-wide polygenic burden per individual in the SUDEP cohort when compared to epilepsy (*P* = 5.7 × 10^− 3^) and non-epilepsy disease controls (*P* = 1.2 × 10^− 3^). The polygenic burden was driven both by the number of variants per individual, and over-representation of variants likely to be deleterious in the SUDEP cohort. As determined by this study, more than a thousand genes contribute to the observed polygenic burden within the framework of this study. Subsequent gene-based association analysis revealed five possible candidate genes significantly associated with SUDEP or epilepsy, but no one single gene emerges as common to the SUDEP cases. Our findings provide further evidence for a genetic susceptibility to SUDEP, and suggest an extensive polygenic contribution to SUDEP causation. Thus, an overall increased burden of deleterious variants in a highly polygenic background might be important in rendering a given individual more susceptible to SUDEP. Our findings suggest that exome sequencing in people with epilepsy might eventually contribute to generating SUDEP risk estimates, promoting stratified medicine in epilepsy, with the eventual aim of reducing an individual patient's risk of SUDEP.

## Highlights

• Increased genome-wide polygenic burden of deleterious genetic variants in people who died of SUDEP
• Some possible candidate genes may carry some of the increased burden of deleterious variants
• If validated, exome sequence analysis may be a tool to estimate genetic susceptibility to SUDEP

## 1 Introduction

Sudden unexpected death in epilepsy (SUDEP) is the commonest cause of epilepsy-related premature mortality (Walczak et al., 2001). The incidence of SUDEP varies from about 1/1000 patient-years in population-based studies (Thurman et al., 2014) up to 6.5/1000 patient-years in cohorts of people with drug-resistant epilepsy unsuitable for surgery (Bell et al., 2010). The precise pathophysiology of SUDEP is unknown: mechanisms may be specific to an individual or shared across individuals, or both. General principles aimed at reducing SUDEP risk, such as seizure control (Ryvlin et al., 2013), should be considered for everyone with epilepsy. The reasons for the effectiveness of such measures, and other preventative strategies (Ryvlin et al., 2013), are not known. Better understanding of the underlying causes of SUDEP is required to establish and target improved preventative strategies.

The cause of SUDEP is likely to be multifactorial, involving underlying genetic susceptibility related to individual epilepsy syndrome (Sakauchi et al., 2011) (of which Dravet Syndrome is the most recognised), brain functional and pathological characteristics (Lhatoo et al., 2010; Bozorgi et al., 2013), uncontrolled generalised tonic–clonic seizures, and the circumstances in which death occurs (e.g. prone position) (Liebenthal et al., 2015). Whilst evidence for genetically-driven mechanisms in SUDEP is provided by familial cases (Hindocha et al., 2008; Kawamata et al., 2010), and animal models (Goldman et al., 2009; Qi et al., 2014; Wagnon et al., 2015), the genetic architecture remains elusive. Substantial genetic heterogeneity is implicated by diverse putative pathophysiologic mechanisms underlying SUDEP (Glasscock et al., 2007; Klassen et al., 2014; Massey et al., 2014).

To elucidate the genetic basis and architecture of SUDEP, we used an unbiased sequencing approach based on whole-exome sequencing data. We examined overall burden and over-representation of deleterious variants in people who died of SUDEP compared to living people with epilepsy and non-epilepsy disease controls.

## 2 Methods

The study was approved by the relevant institutional review boards, accredited regional/national biobanks or international cohorts with ethical frameworks. Details of the difficult issue of sample collection for SUDEP research are given in Supplementary Method 1.

### 2.1 Study Design

**Fig. 1:**
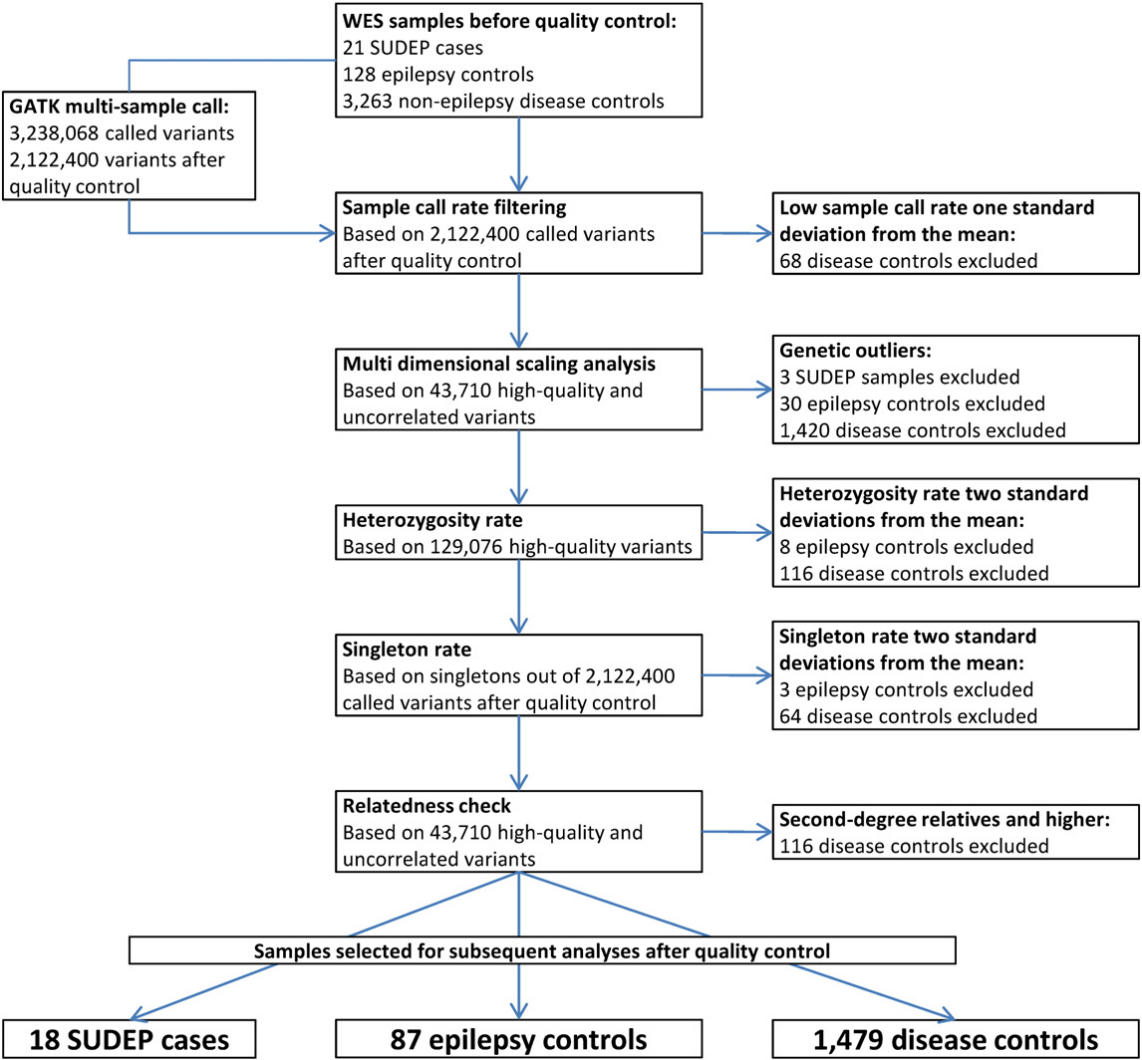
Individual-level quality control flowchart for the SUDEP, epilepsy control, and non-epilepsy disease control samples used in this study. Abbreviations: WES, whole-exome sequencing; SUDEP, sudden unexpected death in epilepsy.

We used whole-exome sequencing (WES) data from 18 people with epilepsy who died of SUDEP and two control cohorts: a group of 87 living people with epilepsy, which we termed ‘epilepsy controls’, and 1479 non-epilepsy ‘disease control’ samples. To ensure data homogeneity, a joint calling strategy, and stringent variant and individual-level quality control (QC) were applied for all WES datasets (Fig. 1 and Supplementary Methods 5–8). Only individuals of white European ancestry were included in subsequent analyses (Supplementary Method 6.2 and Supplementary Fig. 1). We tested the genome-wide burden of rare (or novel) deleterious variants in the SUDEP cohort against both control cohorts separately. Supported by the findings of the genome-wide burden analysis, we sought to identify candidate genes for SUDEP using gene-based association analyses. The study analytic design is outlined in the Supplementary Fig. 2.

### 2.2 Study Participants

The 18 DNA samples from people who had died of SUDEP sometime after DNA donation were selected from DNA archives at the National Hospital for Neurology and Neurosurgery, London (*n* = 8), the Epilepsy Research Centre, Melbourne (*n* = 5), the Royal College of Surgeons in Ireland, Dublin (*n* = 2), the Institute of Life Science, Swansea (*n* = 2), and the Royal Hospital for Sick Children, Glasgow (*n* = 1). The cause of death was classified into definite, probable, or near-SUDEP, according to the most recent proposed system: definite SUDEP required post mortem examination, without an identified toxicological or anatomical cause of death (Nashef et al., 2012). Details of SUDEP cases are given in Supplementary Table 1.

Epilepsy controls (*n* = 87) were patients from the National Hospital for Neurology and Neurosurgery, London (*n* = 71) and the Epilepsy Research Centre, Melbourne (*n* = 16), who had had whole-exome sequencing for other projects and were alive at the time of selection. These controls remain at risk of SUDEP. We applied previous incidence data from a comparable group of people with chronic epilepsy, reporting a SUDEP incidence of 5.9/1000 patient-years (Nashef et al., 1995), to the number of years that our cohort of epilepsy control subjects have already lived with epilepsy (summed minimum epilepsy duration = 2563 years). This suggests that 15/87 would have been expected to have succumbed to SUDEP, whilst, in fact, none have. Thus, the epilepsy control group is enriched with those at lower risk. For all epilepsy cases, we reviewed epilepsy diagnosis (Berg et al., 2010), age at onset of first seizure, presence of intellectual disability (Supplementary Method 2), anti-epileptic drug (AED) treatment, and presence of convulsive or nocturnal seizures over the 12-month period prior to death or latest follow-up. Details of the statistical analyses applied are provided in the Supplementary Method 3.

WES data of disease control samples (pre-QC, *n* = 3,263; post-QC, *n* = 1,479; Supplementary Fig. 2) were obtained from the University College London exomes consortium (UCL-exomes, detailed in the Supplementary Method 4). The disease control samples had no diagnosis of epilepsy or cardiac disease.

### 2.3 Whole-exome Sequencing

All epilepsy samples were sequenced using either Agilent's SureSelect Human All Exon V1 (38 Mb, *n* = 42) and SureSelect Human All Exon V5 (50 Mb, *n* = 56) or Illumina's Nextera Rapid Capture Exome kit (37 Mb, *n* = 16). For the disease control samples, NimbleGen's SeqCap EZ and Illumina's TruSeq Exome capture technology were also used. Sequencing was performed on Illumina HiSeq2500 or GAIIx sequencing systems.

We used a multi-sample joint calling strategy across all SUDEP cases, epilepsy and disease control samples to mitigate problems caused by the heterogeneity of sequence capture kits. One major confound in case–control variant burden analyses can arise when either single-sample calling, or multi-sample calling in different batches, is used to generate the variant calls. Standard practice in single-sample calling is to call non-reference alleles only; calling of all sites is possible but impractical. In order to merge such single-sample calls into one dataset, variants not called in one sample need to be assumed as either homozygous reference, or set to missing. In contrast, multi-sample calling routinely calls homozygous reference genotypes, but only for variants with at least one non-reference allele in the entire sample. Our multi-sample joint calling strategy across all cases and controls as a set enabled us to distinguish between homozygous reference and missing genotypes (Kumar et al., 2014), and provides the basis for standardized QC across aggregated data, essential for case–control designs (Winkler et al., 2014). Details of the variant calling pipeline are given in Sergouniotis et al. (2014) and Supplementary Method 5.

### 2.4 Genome-wide Burden Analysis

Aiming to estimate the burden of mutations at genome-wide level, we chose thresholds for variant QC metrics to maximize specificity over sensitivity, accepting loss of power to detect a significant association in favour of a reduced type I error rate (Supplementary Method 6.1). Individual-level QC filtering generated samples with similar technical sequencing metrics, including overall call rate, singleton rate, and per-individual heterozygosity (Supplementary Method 6.2). After inspection of the population substructure by multidimensional scaling analysis, as implemented in PLINK (Purcell et al., 2007), only samples of clear European ancestry were retained (Supplementary Fig. 1).

For the genome-wide burden analysis of variants in SUDEP, we focussed on variants with the highest likelihood to be pathogenic by selecting rare (minor allele frequency (MAF) ≤ 0.5%), protein-changing variants (Supplementary Method 8). The variant selection procedure for the genome-wide burden analysis is detailed in the Supplementary Method 8.1. We chose this strategy because variant pathogenicity is inversely correlated with the frequency of the non-reference allele in the general population (Coventry et al., 2010), with prediction of variant deleteriousness being more reliable for exonic and splice-site variants than for non-coding variants (Shihab et al., 2015). Using the selected variants, we then assigned to each individual an overall ‘burden score’, calculated by summing the scores for deleteriousness of every selected variant carried per individual, where the deleteriousness of each variant was determined using the Combined Annotation Dependent Depletion method (CADD v1.1) (Kircher et al., 2014, Supplementary Method 7). The CADD method has been proven to achieve high sensitivity in identifying known pathogenic variants. To minimize batch effects between the different WES samples and cohorts, only variants sequenced in more than 80% of the SUDEP cases and the two control cohorts were retained. This strategy was enabled by our joint calling strategy across all cases and controls, and ensured that only variants sequenced in the majority of each of the testing groups were used to calculate the per-individual burden scores. This batch correction method is equivalent to a cross-sample coverage-based correction method, and is not equivalent to the filtering of poorly genotyped variants aimed at removing unreliable genotypes. The threshold of 80% was selected to obtain the lowest variability of all observed per-individual burden scores (Supplementary Method 8.1 and Supplementary Table 5).

We employed the two-tailed Wilcoxon rank-sum test, as implemented in Stata (http://www.stata.com), to compare per-individual burden scores and the number of variants per individual of the SUDEP cases against those of the two control cohorts, as well as epilepsy controls versus disease controls. The threshold for statistical significance was corrected for six tests using the Bonferroni method (two burden tests for three testing groups; α = 8.3 × 10^− 3^).

### 2.5 Gene-based Association Analysis

For the gene-based association analyses, we performed association tests based on comparison of the numbers of non-reference protein-changing variants alleles exclusive to cases versus those exclusive to controls, a well-established unique variant approach (Cohen et al., 2004; Wain et al., 2014). This approach, together with refining based on the predicted deleteriousness (scaled CADD score ≥ 15, Supplementary Method 7), maximizes the power of the gene-based association tests (Ladouceur et al., 2012). Variant and individual-level QC was performed as for the genome-wide burden analysis (Supplementary Method 6). To help dissect out genes more likely conferring risk to SUDEP than to epilepsy, we excluded variants present in the epilepsy control cohort. The variant selection procedure is detailed in the Supplementary Method 8.2.

Empirical data show that the performance of rare variant association methods depends upon the underlying assumption of the relationship between rare variants and complex traits (Ladouceur et al., 2012). We employed a one-tailed burden test of an increased rare allele rate in cases (described in the supplementary information of Purcell et al. (2014)) and the two-tailed C-alpha test (Neale et al., 2011), which allows for risk and protective variants, as implemented in PLINK/SEQ (https://atgu.mgh.harvard.edu/plinkseq). An adaptive permutation procedure was used to assess *P*-values for all association tests (swapping of phenotype label across individuals; genes dropped from further permutation if clearly not associated). We used the PLINK/SEQ estimate of the smallest achievable empirical *P*-value for a gene (*I*-value) to adopt an adjusted Bonferroni correction for multiple testing, by correcting only for the number of genes for which there was power to detect association (*I* < 10^− 3^) (Kiezun et al., 2012). Based on the observed cumulative allele count in the SUDEP cohort for the tested genes, and a Bonferroni-corrected significance threshold, the epilepsy controls did not provide sufficient power to detect associations, and were not used in this component of the study. Confirmatory Sanger sequencing in the SUDEP samples was performed for variants in genes surpassing the adjusted threshold for significance.

### 2.6 Funding

Funding support was provided by Dravet Syndrome UK, the Katy Baggott Foundation, the Epilepsy Society and the Wellcome Trust and EpiPGX (European Union 7^th^ Framework Programme Grant 279062); the Wales Epilepsy Research Network is funded by The National Institute of Social Care and Health Research (NISCHR), Epilepsy Research UK and the Waterloo Foundation; support was provided by the National Health and Medical Research Council of Australia. S.B. was supported by the Polytechnic University of Marche, Italy, with a one-year research fellowship. N.S. was supported by a UCL Impact Studentship, in conjunction with Epilepsy Society. J.N. was supported by the Swiss National Science Foundation-Fellowships for prospective researchers and the SICPA Foundation, Prilly, Switzerland. The UCL Institute of Child Health receives funding as part of GOSH UCL Biomedical Research Centre. This work was partly undertaken at UCLH/UCL, which received a proportion of funding from the Department of Health's NIHR Biomedical Research Centres funding scheme.

## 3 Results

### 3.1 Clinical Phenotype

Eighteen people who died of SUDEP and 87 epilepsy controls were included in subsequent analyses. Demographic and clinical data of these two groups are summarized in Table 1. Eight SUDEP cases fulfilled the criteria for “definite” and 10 were classified as “probable” SUDEP.

The SUDEP group was compared to the living epilepsy controls for the following known clinical risk factors for SUDEP (Hesdorffer et al., 2011; Lamberts et al., 2012): gender, epilepsy syndrome classification, age at first seizure, epilepsy duration, total number of AEDs taken, subjects living alone in the 12-month period before last appointment or death, convulsive or nocturnal seizures in the 12-month period before last follow-up or death. Nominally significant differences were observed only for gender (72% males in SUDEP group versus 41% in living epilepsy controls, *P* = 0.021) and convulsive seizures in the 12-month period before last follow-up or death (present in 72% of SUDEP cases versus 42% of living epilepsy controls, *P* = 0.021). However, none of these differences remained significant after correction of the threshold for statistical significance using the Bonferroni method (for the eight known risk factors stated above; α = 6.3 × 10^− 3^).

Amongst all the epilepsy cases, there was a subset of people with Dravet Syndrome: 30 living Dravet Syndrome cases (26 with, four without, *SCN1A* mutation) and six people with Dravet Syndrome (all with *SCN1A* mutation) and SUDEP, four definite and two probable SUDEP. There was no significant difference in the distribution of the known clinical risk factors for SUDEP or in AED treatment, including exposure to sodium-channel blockers, between people with Dravet Syndrome who died of SUDEP and the living Dravet Syndrome cases, after correction for multiple testing (Supplementary Table 2). Details of *SCN1A* mutations are presented in the Supplementary Tables 3 and 4.

### 3.2 Genome-wide Burden of Rare Deleterious Variants

After individual-level QC, 18 SUDEP, 87 epilepsy, and 1,479 disease control samples were included in subsequent analyses (Fig. 1). Variants with at least one non-reference allele in any of the SUDEP, epilepsy, and disease control samples were selected for the analyses (*n* = 89,512; Supplementary Fig. 2). The 89,512 variants represented 1707 genes of the human reference genome with non-reference alleles in the SUDEP samples, 5464 genes with non-reference alleles in the epilepsy controls, and 13,887 genes with non-reference alleles in the disease controls (union = 13,999 genes). Details of coverage are given in the Supplementary Result 10.

**Fig. 2:**
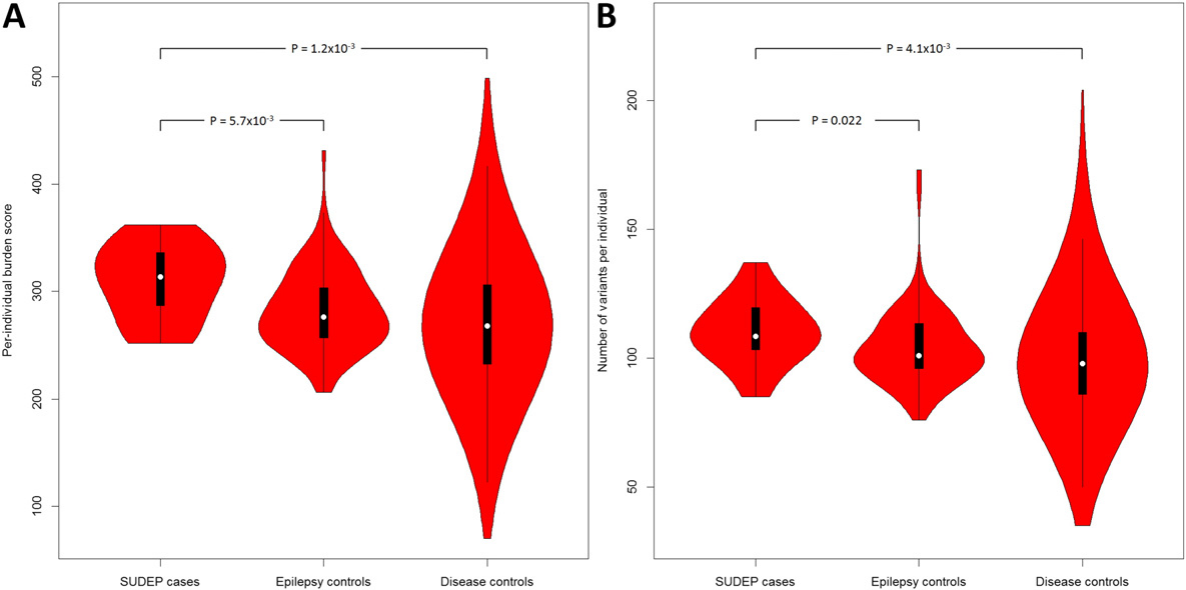
Violin plots of the burden score and variant number per individual. Plotted are the per-individual burden scores (A) and the number of variants per individual (B) of each test group. A violin plot is a box plot with the width of the box proportional to the estimated density of the observed data (proportion of cases with given ordinate value). The maximum density of the group-specific data distribution is indicated by the largest width of the violins. The density trace is plotted symmetrically to the left and the right of the box plot for better visualization. All violins have the same fixed maximum width. The white dot is the median, the thick black vertical bar represents the interquartile range (IQR), and the thin black vertical bar represents 95% confidence intervals.

We observed a significantly increased genome-wide burden score per individual in the SUDEP cohort when compared to epilepsy (*P* = 5.7 × 10^− 3^) and non-epilepsy disease controls (*P* = 1.2 × 10^− 3^) (Table 2; Fig. 2, Supplementary Table 6). The number of variants per individual showed suggestive over-representation against the epilepsy controls (*P* = 0.022), and significant over-representation against disease controls (*P* = 4.1 × 10^− 3^) (Table 2, Fig. 2). Although there was also a significant difference in the number of variants between the two control cohorts (*P* = 6.1 × 10^− 3^), the genome-wide burden score did not differ. This genome-wide burden suggests an extensive polygenic contribution to SUDEP causation.

Post hoc analysis removing all post-QC *SCN1A* variants showed that the genome-wide burden was not biassed by the enrichment of both the SUDEP and the epilepsy cohorts with Dravet Syndrome patients bearing *SCN1A* mutations (comparison against epilepsy controls: *P* = 6.3 × 10^− 3^; disease controls: *P* = 1.4 × 10^− 3^).

### 3.3 Gene-based Association of Unique Deleterious Variants

Gene-based association tests were performed for all genes with at least one non-reference allele in either SUDEP cases or disease controls (373 genes in the SUDEP cases; 10,319 genes in the disease controls; union = 10,405). The threshold for statistical significance was corrected for 32 tests using the adjusted Bonferroni method (two association tests for 16 genes with *I* < 10^− 3^; α = 1.56 × 10^− 3^). Five genes harbouring Sanger-confirmed variants were significantly associated with SUDEP when compared to the 1479 disease controls (Table 3). The most strongly associated gene was *SCN1A* (C-alpha *P* = 1.61 × 10^− 4^), followed by *LGI1* (lowest *P* = 3.12 × 10^− 4^), *SMC4* (lowest *P* = 5.39 × 10^− 4^), *COL6A3* (lowest *P* = 7.27 × 10^− 4^), and *TIE1* (lowest *P* = 1.48 × 10^− 3^). Sanger sequencing failed to confirm one of two variants of the *PIK3C2A* gene. We note that we considered only *SCN1A* variants that passed the same QC filtering applied to every other WES-derived variant in any other gene. Coverage statistics for the WES target intervals within the genes are given in Table 3.

## 4 Discussion

SUDEP is the most devastating outcome in epilepsy. Whilst a number of risk factors and terminal pathophysiological phenomena have been determined, the cause of SUDEP remains unknown. There appear to be environmental risk factors, and evidence for genetic susceptibility. Given evidence for heterogeneity of genetic risk, we proposed that genetic risk is spread across the genome. We show that, in people who have succumbed to SUDEP, there is a higher burden of deleterious genetic variants, with a higher cumulative deleteriousness score, compared to the burden in people with epilepsy who had not succumbed to SUDEP, and compared to the burden in people without epilepsy. Gene-based analysis in this group of SUDEP cases identifies some possible candidate genes that may carry some of the excess burden in this small sample. Our results provide further evidence for genetic susceptibility to SUDEP.

The identified genetic susceptibility is spread across the genome. Deleterious variants exclusively present in the exomes of this SUDEP group were found in 373 genes in the human genome. One of these genes is associated with cardiac arrhythmia (*CACNB2*, Supplementary Table 5 and Supplementary Result 11). No other genes previously implicated in sudden cardiac death emerged. There are some genes that in our small SUDEP group appear overburdened (*n* = 5), but no one single gene, nor one single pathway, emerges as common to all SUDEP cases. Our findings require confirmation in an independent cohort. Taking the known genetic heterogeneity of syndromes associated with a higher risk of SUDEP together with our findings, we suspect that there is indeed not one culpable pathway or gene set for SUDEP. Studies of other SUDEP case groups might identify additional sets of risk variants. Even though observational studies report mutations in SUDEP in candidate genes, we note that single candidate gene studies have not revealed a robust association with SUDEP in humans (Bagnall et al., 2014). We propose that an overall increased burden of deleterious variants in a highly polygenic background is important in rendering a given individual more susceptible to SUDEP.

Some deleterious variants we have identified may per se contribute to, or be the cause of, the epilepsy, as well as increasing SUDEP risk. This may be the case, for example, for some *SCN1A* mutations that were already known in the Dravet Syndrome cases and held responsible for the condition. It is unlikely that these single mutations were solely responsible for SUDEP in these cases, as SUDEP is not universal in Dravet Syndrome, although a higher frequency of SUDEP is well recognised to occur (Sakauchi et al., 2011). Notably, *SCN1A* emerged as a burdened gene even when considering only WES-derived variants that passed variant selection. The exclusion of many *SCN1A* variants considered causal before QC is due to our strict and conservative QC, emphasising specificity above sensitivity. Nevertheless, *SCN1A* still emerged as a burdened gene. A possible dual role in both disease and SUDEP causation may apply to variants in other genes as well.

SUDEP genetics is an important area, and we must acknowledge limitations to our study. The number of individuals who succumbed to SUDEP is small. Whilst there are new efforts to address this problem, to date case recognition and ascertainment (Smithson et al., 2014), collection of suitable samples and difficulties in obtaining WES data from certain types of material, have hampered progress and limited numbers. Dravet Syndrome is over-represented in both SUDEP and epilepsy control groups compared to the general population of people with epilepsy, though we note that SUDEP is also more common in people with Dravet Syndrome than in the overall population of people with epilepsy. Whilst we cannot exclude the possibility that any individual in our epilepsy control might succumb to SUDEP in the future, none has yet despite an expectation that a proportion might have been expected to do so, such that our epilepsy control group is enriched with those at lower risk of SUDEP. Although a significantly higher prevalence of male gender and convulsive seizures in the 12-month period before last follow-up or death was observed in the SUDEP cases compared to the epilepsy controls, these differences do not survive correction for multiple comparisons. Nevertheless, the differences merit some discussion. Male gender has been associated with a 1.4-fold increased risk for SUDEP in a combined analysis of case–control studies (Hesdorffer et al., 2011). Other previous studies did not confirm this association (Walczak et al., 2001; P-Codrea Tigaran et al., 2005; Vlooswijk et al., 2007) and more recently a mouse model of SUDEP did not show significantly different susceptibility to seizure-induced respiratory arrest between males and females (Faingold and Randall, 2013). Overall, the difference in the proportion of males in the SUDEP and epilepsy control groups may therefore not be biologically relevant, and is not in any case statistically significant after correction for multiple comparisons. The difference in convulsive seizure frequency between the SUDEP and epilepsy control groups is also not significant after correction for multiple comparisons, but it is interesting to speculate whether genome-wide burden of deleterious variants is an explanation that might underlie this epidemiologically-derived risk factor, tying epilepsy severity into genomic burden.

The burden test used in our genome-wide burden analysis is sensitive to linkage disequilibrium (increased type I error rate). The comparatively small epilepsy control dataset may mean that we have not adequately filtered out deleterious variants related to epilepsy causation rather than to SUDEP in our gene-based association analyses only. The associated genes may contribute to both epilepsy and SUDEP causation. We used different tests for the gene-based association analyses: replication of the results in an independent sample using the same statistical tests is needed. Our strategy focuses on deleterious rare variants: other types of genetic variant may also influence SUDEP risk. We did not undertake functional studies, but such studies are likely to prove extremely challenging, requiring not only construct complexity or multiple knock-ins, but also a whole animal model, as agonal changes in SUDEP typically occur outside the brain.

The finding of genome-wide increased burden of deleterious variants, rather than the individual genetic results, needs replication. If substantiated, these results provide scope for individualised risk estimates of SUDEP in people with epilepsy, with direct consequences for use of current strategies to reduce risk through improved seizure control or environmental measures, and may also assist with recurrence risk estimation in affected family members. The results highlight the value of exome sequencing in people with epilepsy: one test can provide insights into possible genetic causation, pharmacogenomic variants and outcome risk estimation. Overall, the findings provide new perspectives into SUDEP.

## Author Contributions

CL and SMS designed the study. SB, KH, NS, JN, JW, RE, ER, MOR, WOP, RHT, SKC, ND, JMM, SM, LGS, SFB, LN, SMZ, MIR, GLC, JWS, EHu, JHC, IES, and SMS contributed patient samples and collected clinical and genetic data. PG, EHä, and VP conducted and supervised exome sequencing and variant calling. BM and LHH performed Sanger sequencing. CL and SB analysed and interpreted data. AP and SMS supervised the study. CL, SB and SMS wrote the manuscript. All authors critically reviewed and approved the manuscript.

## Conflicts of Interest

J.N. has received personal/institutional honoraria from UCB and Pfizer. L.N. has received personal/institutional honoraria from UCB and Eisai and received systems for clinical use (external trigeminal stimulation as treatment for epilepsy) at her institution from NeuroSigma, Inc. S.M.Z. has received research funding or personal/institutional honoraria from UCB, GW Pharma and Brabant Pharma. M.I.R. has received non-conditional research funding from UCB. G.L.C. has received research funding from UCB and personal honoraria from Eisai Inc. J.W.S. reports grants/grants pending from GSK, Eisai, WHO, NEF. J.W.S. reports personal/institutional honoraria for lectures, including service on speakers bureaus, from Lundbeck, Teva, Eisai, UCB, and having board membership and a consultancy relationship on drug development. J.W.S.'s current position is endowed by the Epilepsy Society. J.W.S. is member of the Editorial Board of the Lancet Neurology. J.W.S. receives research support from the Marvin Weil Epilepsy Research Fund. J.H.C. holds an endowed Chair through the University College, London. She has sat on Advisory Panels for Eisai and Viropharma for which remuneration has been paid to her department. She has received grants for research from GW Pharma and Vitaflo. She works as Clinical Advisor to the National Children's Epilepsy Surgery Service for which remuneration is made to her department. I.E.S. reports grants from NHMRC, grants from NIH, during the conduct of the study; she is on the Editorial Board of Annals of Neurology, Epileptic Disorders and Neurology; she reports personal fees from UCB, Athena Diagnostics, Transgenomics, GlaxoSmithKline, Biocodex, Sanofi, outside the submitted work. In addition, I.E.S. has a patent on Diagnostic and Therapeutic Methods for EFMR (Epilepsy and Mental Retardation Limited to Females) with royalties paid. S.M.S. has received research funding or personal/institutional honoraria from UCB, GSK and Eisai Inc, and research support from UCB and has an academic collaboration with Complete Genomics. All other authors declare no competing interests.

## Figures and Tables

**Table 1:** Demographic and clinical features of SUDEP cases and living epilepsy controls.

		SUDEP cases (*n* = 18)	Living adult epilepsy controls (*n* = 87)	Uncorrected *P*-value (test)
Mean age at last recorded follow-up/death, years (SD)		29 (18)	35 (16)	0.198 (t-test)
Gender, *n* = male (%)		13 (72)	36 (41)	0.021 (Fisher's exact)
Epilepsy syndrome classification, *n* (%)	DS	6 (33)	30 (35)	0.423 (Pearson *χ*^2^)
Focal S.	5 (28)	25 (29)
Focal U.	4 (22)	7 (8)
GGE	1 (6)	14 (16)
UE	2 (11)	11 (13)
Median age at first seizure occurrence, years (IQR)		2.5 (0.9–13)	2 (0.5–7)	0.332 (Wilcoxon rank-sum)
Median epilepsy duration, years (IQR)		20 (10–38)	30 (19–43)	0.086 (Wilcoxon rank-sum)
Intellectual disability, *n* (%)^a^		10 (56)	38 (45)	0.402 (Pearson *χ*^2^)
Total number of AEDs taken, median (IQR)		8 (5–11)	8 (4–10)	0.997 (Wilcoxon rank-sum)
Subject living alone in the 12-month period before last follow-up/death, *n* (%)^a^		2 (12)	6 (7)	0.617 (Fisher's exact)
Convulsive seizures in the 12-month period before last follow-up/death, *n* (%)^a^		13 (72)	35 (42)	0.021 (Fisher's exact)
History of nocturnal seizures in the 12-month period before last follow-up/death, *n* (%)^a^		5 (33)	34 (42)	0.775 (Fisher's exact)

The Bonferroni method was applied to correct for the following known risk factors for SUDEP: gender, epilepsy syndrome classification, age at first seizure, epilepsy duration, total number of AEDs taken, subjects living alone in the 12-month period before last appointment or death, convulsive or nocturnal seizures in the 12-month period before last follow-up or death. The threshold for statistical significance after Bonferroni correction was set to α = 6.3 × 10^− 3^. Abbreviations: SUDEP, sudden unexpected death in epilepsy; DS, Dravet Syndrome; Focal U., Focal unknown aetiology; Focal S., Focal symptomatic; GGE, Genetic Generalised Epilepsy; UE, Unclassified Epilepsy (Berg et al., 2010).

a Missing data: intellectual disability (*n* = 2); subject living alone in the 12-month period before last follow-up/death (*n* = 3); convulsive seizures in the 12-month period before last follow-up/death (*n* = 3); history of nocturnal seizures in the 12-month period before last follow-up/death (*n* = 8).

**Table 2:** Genome-wide burden analysis results based on 89,512 quality-control filtered, protein-changing, and rare variants.

	SUDEP patients			Epilepsy controls			Disease controls			Wilcoxon rank-sum test *P*-values^a^		
(*n* = 18)			(*n* = 87)			(*n* = 1479)		
M	Mdn	IQR (Q1–Q3)	M	Mdn	IQR (Q1–Q3)	M	Mdn	IQR (Q1–Q3)	SUDEP vs. epilepsy controls	SUDEP vs. disease controls	Epilepsy controls vs. disease controls
Test groups										18 vs. 87	18 vs. 1479	87 vs. 1479
Per-individual burden scores	309.2	313.3	54.3 (284–338)	282.7	276.3	47.2 (257–304)	270.3	268.4	73.5 (233–306)	5.7 × 10^− 3^	1.2 × 10^− 3^	0.023
N. of variants per individual	110.2	108.5	18 (102–120)	104.1	101	18 (96–114)	99.29	98	24 (86–110)	0.022	4.1 × 10^− 3^	6.1 × 10^− 3^

Post hoc analysis excluding SCN1A variants^b^												
Per-individual burden scores	308.2	312.6	54.3 (284–338)	282.2	276.3	46.5 (256–303)	270.3	268.4	73.5 (233–306)	6.3 × 10^− 3^	1.4 × 10^− 3^	0.028

Threshold for statistical significance after Bonferroni correction was set to α = 8.3 × 10^− 3^. Abbreviations: SUDEP, sudden unexpected death in epilepsy; M, mean; Mdn, median; IQR, interquartile range; Q1, lower (first) quartile; Q3, upper (third) quartile; N., number.

a All *P*-values are two-tailed.

b Post hoc analysis excluding 31 *SCN1A* variants present in any of the testing groups.

**Table 3:** Gene-based association analysis results.

Gene	Cytoband GRCh37	Cumulative non-reference allele count (cumulative minor allele frequency, %)^a^			Mean average coverage			Percent of target bases with 10 × or greater coverage			18 SUDEP cases vs. 1479 disease controls	
SUDEP cases	Epilepsy controls	Disease controls	SUDEP cases	Epilepsy controls	Disease controls	SUDEP cases	Epilepsy controls	Disease controls	Burden *P*-value⁎⁎⁎	C-alpha *P*-value⁎⁎⁎
(*n* = 18)	(*n* = 87)^b^	(*n* = 1,479)
*SCN1A*	2q24.3	2 (5.56)	18 (11.19)	4 (0.16)	51 ×	44 ×	75 ×	87%	81%	89%	1.21 × 10^− 4^	1.61 × 10^− 4^
*LGI1*	10q23.33	2 (5.56)	0 (0)	2 (0.08)	90 ×	67 ×	45 ×	80%	78%	68%	3.12 × 10^− 4^	3.12 × 10^− 4^
*PIK3C2A*	11p15.1	2 (5.56)	1 (0.61)	1 (0.04)	81 ×	58 ×	69 ×	93%	90%	87%	3.12 × 10^− 4^	3.34 × 10^− 4^
*SMC4*	3q25.33	2 (5.56)	0 (0)	1 (0.05)	92 ×	57 ×	36 ×	79%	73%	63%	5.39 × 10^− 4^	5.39 × 10^− 4^
*COL6A3*	2q37.3	2 (5.56)	0 (0)	5 (0.19)	77 ×	63 ×	51 ×	83%	84%	76%	7.27 × 10^− 4^	7.27 × 10^− 4^
*TIE1*	1p34.2	2 (5.56)	0 (0)	4 (0.14)	85 ×	58 ×	37 ×	71%	70%	59%	1.48 × 10^− 3^	2.01 × 10^− 3^

Shown are six genes significantly associated with SUDEP when compared to the 1479 disease controls. *P*-values surpassing the Bonferroni-corrected threshold for significance (α = 1.56 × 10^− 3^) are highlighted in grey. Sanger sequencing failed to confirm one variant for the *PIK3C2A* gene shown in red; the gene is not considered as associated with SUDEP. Abbreviations: SUDEP, sudden unexpected death in epilepsy; GRCh37, Genome Reference Consortium Human genome build 37.

a Cumulative counts and frequencies are the summed counts and frequencies of the non-reference alleles.

b Cumulative counts and frequencies for the epilepsy controls are given for comparison only. Association tests were not performed, as explained in the text.

⁎⁎⁎ All *P*-values are based on adaptive permutations. Burden *P*-values are one-tailed; C-alpha *P*-values are two-tailed. Genes are ranked by the C-alpha *P*-value.
