# Supplementary material for: Genome-wide Polygenic Burden of Rare Deleterious Variants in Sudden Unexpected Death in Epilepsy
Source: eBioMedicine. 2015 Jul 10;2(9):1063–70. doi: 10.1016/j.ebiom.2015.07.005 (PMC4588398; doi:10.1016/j.ebiom.2015.07.005)
Supplement: Supplementary file 1 — Supplementary Material. [file mmc1.docx]

**Supplementary Material for:**

Leu C, Balestrini S, et al. (2015) Genome-wide polygenic burden of rare deleterious variants in sudden unexpected death in epilepsy.

Corresponding Author:

Prof. Dr. Sanjay M Sisodiya

Department of Clinical and Experimental Epilepsy

UCL Institute of Neurology

33 Queen Square

London WC1N 3BG

United Kingdom

Tel: +44 (0) 20 3448 8612

Fax: +44 (0) 20 3448 8615

Email: [s.sisodiya@ucl.ac.uk](mailto:s.sisodiya@ucl.ac.uk)

**Supplementary Methods**

1. Sample collection
2. Intellectual Disability Assessment
3. Statistical analysis of clinical phenotype
4. University College London exomes consortium
5. Whole-exome sequencing
6. Quality control (QC)
   1. Variant QC
   2. Individual-level QC
7. Prediction of variant deleteriousness
8. Variant Annotation and filtering
   1. Variant filtering for genome-wide burden analyses only
   2. Variant filtering for gene-based burden analyses only
9. Variant validation for the gene-based burden analyses
   1. Sanger sequencing

**Supplementary Results**

1. Supplementary Table 1. Details of the 18 SUDEP cases
2. Supplementary Table 2. Demographic and clinical features of Dravet Syndrome cases comparing those who died of SUDEP with living cases
3. Supplementary Table 3. *SCN1A* mutations identified prior to whole exome sequencing in the Dravet Syndrome patients who died of SUDEP
4. Supplementary Table 4. *SCN1A* mutations identified prior to WES in the living Dravet Syndrome cohort
5. Supplementary Table 5. Coefficients of variation of the cumulative per-individual burden scores after applying different methods for batch correction
6. Supplementary Figure 1. Multidimensional scaling analysis
7. Supplementary Figure 2. Study design and variant filtering flowchart
8. Supplementary Figure 3. Notched boxplot of the per-individual burden scores
9. Supplementary Figure 4. Notched boxplot of the number of variants per individual
10. Supplementary Result 10: Whole-exome sequencing coverage
11. Supplementary Result 11: Deleterious singleton variants in genes implicated in cardiac death or epilepsy causation
12. Supplementary Table 6. Burden scores and variant numbers for the SUDEP and epilepsy control samples
13. Supplementary Table 7. List of all 373 genes with at least one non-reference variant in the SUDEP cases

**Supplementary Methods**

**1. Sample collection**

Collection of SUDEP samples for genetic studies provides an unusual level of challenge. SUDEP cannot be predicted, so there is no ‘target’ population. Collection after SUDEP is difficult to systematize, as by definition death is unexpected and cannot be anticipated, leading to logistic difficulties of obtaining material after death (Smithson *et al.*, 2014).

**National Hospital for Neurology and Neurosurgery**

At the National Hospital for Neurology and Neurosurgery, DNA samples have been collected from thousands of patients for an approved broad study of epilepsy genetics and pharmacogenomics. By chance, some of the individuals who gifted DNA samples sadly succumbed to SUDEP. These were the samples used in this study.

**Wales Epilepsy Research Network (WERN), Swansea University**

WERN has an accredited epilepsy BioBank with 3,000 samples including epilepsy families and specific cohorts and has IRAS approval for the infrastructure project. Samples submitted to this study were gifted by consent prior to the tragic SUDEP event. We thank the families for their post-SUDEP advocacy of the research and their positive bravery in the search for the cause.

**Royal College of Surgeons in Ireland**

At Beaumont Hospital / RCSI, Dublin, DNA samples have been collected from over 1,500 of patients with different types of epilepsy for an approved broad study of epilepsy genetics and pharmacogenomics. Using our epilepsy electronic patient record database we identified two patients from whom DNA had been collected and who had died of SUDEP. These samples were used in this study.

**Epilepsy Research Centre, Melbourne**

The Epilepsy Genetics Research Program at the Epilepsy Research Centre, Austin Health, University of Melbourne, has been fortunate to have many thousands of participants with epilepsy provide DNA samples over 25 years. Sadly, some participants have subsequently passed away from SUDEP. We thank our participants and their families for their ongoing support of our research, especially following such a tragic event.

**Royal Hospital for Sick Children**

At the Royal Hospital for Sick Children, Glasgow DNA samples have been collected from patients for clinical testing of epilepsy genes. A cohort of patients with *SCN1A*-related epilepsy was enrolled in a research project. By chance some of the individuals who gifted DNA samples sadly succumbed to SUDEP. These were the samples used in this study.

**2. Intellectual Disability Assessment**

All available clinical information for epilepsy cases was reviewed. Presence of intellectual disability was defined as an intelligence quotient < 70 from a previous psychometric assessment, with onset under 18 years of age, or systematic mention of “learning/intellectual disability” or “mental retardation” in the medical notes.

**3. Statistical analysis for clinical phenotype**

To compare the clinical features of the SUDEP cases and epilepsy controls, two-sample t-test or Wilcoxon rank-sum test were used for continuous variables showing Gaussian or non-Gaussian distributions, respectively. Pearson χ^2^ test or Fisher’s exact test, as appropriate according to the sample size in any of the cells of contingency tables, were used to compare categorical variables. Two-tailed *P*-values of the Fisher’s exact test were calculated. Raw *P*-values are given in Table 1 and in Supplementary Table 2. Bonferroni correction of the nominal threshold for significance of 0.05 was subsequently applied. We had less than 10% missing data and we performed sensitivity analyses of deviations from the assumption of missing at random. Sensitivity analyses showed that missing data did not cause any bias to our results. We therefore present results for subjects with complete data. The amount of missing data for each clinical variable is noted in the footnotes of Table 1 and Supplementary Table 2. Data were analyzed using Stata (<http://www.stata.com>).

**4. University College London exomes consortium**

The University College London exomes consortium (UCL-exomes) is a consortium of researchers within University College London (London, UK) designed to aggregate raw read-level data from multiple exome sequencing projects in order to facilitate case-control association studies. At the time of this study, the UCL-exomes dataset included 3,412 samples (21 SUDEP, 128 epilepsy, and 3,263 non-epilepsy disease samples). The 3,263 non-epilepsy disease samples had no diagnosis of cardiac disease.

**5. Whole-exome sequencing**

Whole-exome sequencing for all 3,412 UCL-exomes samples (including 21 SUDEP and 128 epilepsy controls; pre-QC) was performed using Agilent, NimbleGen, and Illumina sequence capture with Illumina sequencing on HiSeq or GAIIx instruments. Fastq files were aligned with Novoalign (<http://www.novocraft.com>) against the reference human genome (GRCh37). Duplicate read removal, format conversion, and indexing were performed using Picard (<http://broadinstitute.github.io/picard>). The Genome Analysis Toolkit (GATK) (McKenna *et al.*, 2010) was used for variant calling, with Variant Quality Score Recalibration (VQSR) and separate models for SNPs and indels, following best practice (DePristo *et al.*, 2011; Van der Auwera *et al.*, 2013). Multi-sample variant calling was performed using the GATK HaplotypeCaller on 3,412 samples of the UCL-exomes consortium. We used the union of the different target regions for variant calling, +/- 100 base-pairs on each side of the target regions. Read depth was excluded from the recalibration model because of the large read depth variability generated by the heterogeneous capture kits used in the multiple studies aggregated in the UCL-exomes cohort.

**6. Quality control (QC)**

**6.1 Variant QC**

The following QC thresholds were applied for all variant calls using VCFtools (Danecek *et al.*, 2011): (i) GATK truth sensitivity 99.5% for single nucleotide variants (SNVs) and 95% for indels; (ii) genotype quality (GQ) ≥ 20 for homozygous and ≥ 40 for heterozygous calls; (iii) maximum two alleles; (iv) sample read depth (DP) of high-quality reads ≥ 10; (v) Hardy-Weinberg equilibrium (HWE) with *P* > 10^-20^; (vi) call rate (CR) ≥ 1% in the 3,412 samples of the multi-sample call. 2,122,400 out of 3,238,068 variants called in 3,412 samples passed the QC thresholds.

**6.2** **Individual-level QC**

To minimize the type I error rate on rare variant burden analyses (Luedtke *et al.*, 2011), only individuals of white European ancestry were included (self-reported, and by inspecting the first 20 coordinates of a multidimensional scaling analysis (MDS), Supplementary Fig. 1). Related individuals with a proportion of alleles shared identically by descent according to second-degree relatives and higher (π-hat ≥ 25%) were excluded. In addition, extensive sample QC was applied to ensure technical (sequencing assay) homogeneity of the remaining samples. Samples were excluded for the following criteria: (i) low sample CR one standard deviation (SD) from the mean; (ii) singleton rate two SD from the mean; (iii) heterozygosity rate two SD from the mean. Sample QCs were performed using PLINK (Purcell *et al.*, 2007). For MDS, per-individual heterozygosity and pairwise relatedness estimation, we used a trimmed set of variants (autosomal variants only, call rate ≥ 90%, minor allele frequency (MAF) ≥ 0.1%, and linkage disequilibrium r^2^ < 0.5 for the MDS only). Singleton rates were calculated using PLINK/SEQ (<https://atgu.mgh.harvard.edu/plinkseq>). Out of 21 SUDEP samples, 18 passed the individual-level QC; 87 out of 128 epilepsy controls, and 1,479 out of 3,263 UCL-exomes non-epilepsy disease controls (Fig. 1) passed the same stringent QC.

**7. Prediction of variant deleteriousness**

We used the recently published Combined Annotation Dependent Depletion method (CADD) (Kircher *et al.*, 2014), to predict the deleteriousness of variants. The CADD framework integrates multiple annotations into one metric, with the advantage that it allows the ranking of every variant, based on the predicted deleteriousness, among all GRCh37/hg19 reference SNVs (~8.6 billion). We used pre-scored files provided for download (version 1.1) and the CADD web interface to generate the CADD raw and scaled scores for all sequenced and QC-filtered variants (*n* = 2,122,400). The CADD raw scores were used to generate the cumulative per-individual burden scores for the genome-wide burden analysis. Scaled CADD scores were used to select the most deleterious variants (scaled CADD score ≥ 15; median value for all possible canonical splice site changes and non-synonymous variants) for the gene-based association analyses.

**8. Variant Annotation and filtering**

We used ANNOVAR (Wang *et al.*, 2010) to select variants based on the following criteria: (i) protein-changing variants according to the hg19 Reference Sequence (RefSeq) gene transcripts (UCSC Genome Browser, <http://genome.ucsc.edu>) (stop-gain/loss, splice-site variants within 2bp of an exon-intron boundary, frameshift/non-frameshift indels, and non-synonymous variants); (ii) not located within segmental duplications, to avoid artifacts due to paralogous sequence variation (Bailey *et al.*, 2001; Wang *et al.*, 2010). Out of 2,122,400 post-QC variants, 402,181 were classified as protein-changing. Out of 402,181 protein-changing variants, 203,089 variants, present with at least one non-reference allele in the samples which passed individual-level QC (18 SUDEP patients, 87 epilepsy controls, 1,479 UCL-exomes non-epilepsy disease controls), were selected for subsequent analyses. ANNOVAR was also used for subsequent filtering based on the MAF.

**8.1 Variant filtering for genome-wide burden analyses only**

The selected 203,089 protein-changing variants were filtered to be rare, defined by a MAF ≤ 0.5% (arbitrary, but commonly-used, threshold to define a rare variant (Tennessen *et al.*, 2012; Hunt *et al.*, 2013)) according to three publicly-available datasets: Exome Aggregation Consortium (ExAC) v0.2 non-Finnish Europeans (*n* = 34,427), NHLBI-ESP European-Americans (*n* = 4,300), and 1000genomes October 2014 Europeans (*n* = 503). Out of 203,089 protein-changing variants, 166,603 were selected as protein-changing and rare (or novel) variants for the genome-wide burden analyses.

Additional variant QC was applied for the genome-wide analyses to mitigate batch effects. Variant missing data rates were calculated using VCFtools in the SUDEP, epilepsy, and disease control samples separately. The generated missing data rates were used as custom databases for the annotation with ANNOVAR. Subsequently, only variants sequenced in more than 80% of each test group were retained. This method was more efficient in removing sequencing batch effects than a correction method based on target interval mean coverage of the three groups, as indicated by the coefficients of variation of the cumulative per-individual burden scores (Supplementary Table 5). A higher sequencing threshold for filtering did not lead to a lower variance of the values around their mean. Finally, 89,512 variants were included in the analysis.

**8.2 Variant filtering for gene-based association analyses only**

The selected 203,089 protein-changing variants were filtered to be novel according to (i.e. not present in) the ExAC v0.2 non-Finnish Europeans, NHLBI-ESP European-Americans, and 1000genomes October 2014 Europeans. Variants present in the epilepsy control cohort were also excluded. Following our unique variant strategy, we filtered the remaining variants to be exclusive to the SUDEP or exclusive to the disease control samples. We then selected the most deleterious variants, following the recommendations of the prediction software used (scaled CADD score ≥ 15; median value for all possible canonical splice site changes and non-synonymous variants).

VCFtools was used to generate the filtered datasets for association testing.

**9.** **Variant validation for the gene-based association analyses**

Aligned sequence data for 12 variants selected from six genes significantly associated with SUDEP in the gene-based association analyses and six singletons observed in genes implicated in either cardiac death or epilepsy, were visually inspected using the IGV browser (Robinson *et al.*, 2011).

**9.1 Sanger sequencing**

Confirmatory Sanger sequencing in the SUDEP samples was performed for the variants which passed the visual inspection. Primers for the regions of interest were designed using primer3 software (http://bioinfo.ut.ee/primer3/). Polymerase chain reaction (PCR) was performed according to the optimal conditions of the designed primers. PCR products were purified using ExoSAP-IT (Affymetrix, Santa Clara, CA, USA), and sequenced using BigDye v. 3.1 (Applied Biosystems) on an ABI3730xl automated DNA sequencer.

**Supplementary Results**

**Supplementary Table 1. Details of the 18 SUDEP cases.**

| **ID** | **Gender** | **Age of death** | **Epilepsy syndrome** | **SUDEP** |
| --- | --- | --- | --- | --- |
| 4 | F | 7 | DS | Definite |
| 5 | F | 11 | DS | Definite |
| 6 | M | 6 | DS | Definite |
| 1 | M | 12 | DS | Definite |
| 37 | F | 42 | Focal S. | Definite |
| 39 | M | 20 | Focal S. | Definite |
| 48 | M | 18 | Focal U. | Definite |
| 38 | F | 32 | GGE | Definite |
| 3 | F | 3 | DS | Probable |
| 2 | M | 20 | DS | Probable |
| 41 | M | 44 | Focal S. | Probable |
| 46 | M | 46 | Focal S. | Probable |
| 47 | M | 67 | Focal S. | Probable |
| 43 | M | 35 | Focal U. | Probable |
| 40 | M | 38 | Focal U. | Probable |
| 45 | M | 40 | Focal U. | Probable |
| 44 | M | 32 | UE | Probable |
| 42 | M | 56 | UE | Probable |

Abbreviations: ID = identification number, SUDEP = sudden unexpected death in epilepsy, M = male, F = female, DS = Dravet Syndrome, Focal U. = Focal unknown aetiology, Focal S. = Focal symptomatic, GGE = Genetic Generalised Epilepsy, UE = Unclassified Epilepsy (Berg *et al.*, 2010).

**Supplementary Table 2. Demographic and clinical features of Dravet Syndrome cases comparing those who died of SUDEP with living cases.**

Bonferroni method was applied to correct for exposure to each AED and for the following known risk factors for SUDEP: gender, age at first seizure, epilepsy duration, total number of AEDs taken, subjects living alone in the 12-month period before last appointment or death, convulsive or nocturnal seizures in the 12-month period before last follow-up or death. Threshold for statistical significance after Bonferroni correction was set to α = 0.002.

|  | **Dravet Syndrome cases who died of SUDEP** | **Living Dravet Syndrome cases** | **Uncorrected** | **Test** |
| --- | --- | --- | --- | --- |
|  | ***n* = 6** | ***n* = 30** | ***P*-value** |  |
| Mean age at last recorded follow-up/death, years (SD) | 10 (6) | 36 (11) | <0.001 | t-test |
| Gender, n male (%) | 3 (50) | 12 (40) | 0.677 | Fisher’s exact |
| Median age at first seizure occurrence, years (IQR) | 0.7 (0.4-0.9) | 0.6 (0.5-0.7) | 0.732 | Wilcoxon rank-sum |
| Mean epilepsy duration, years (SD) | 9 (6) | 35 (11) | <0.001 | t-test |
| Intellectual disability | 6 (100) | 29 (97) | 1 | Fisher’s exact |
| Total number of AEDs taken, median (IQR) | 8 (5-10) | 10 (9-11) | 0.095 | Wilcoxon rank-sum |
| Exposure to acetazolamide (%) | 1 (17) | 8 (27) | 1 | Fisher’s exact |
| Exposure to carbamazepine (%) | 4 (67) | 29 (97) | 0.066 | Fisher’s exact |
| Exposure to clobazam (%) | 5 (83) | 20 (67) | 0.643 | Fisher’s exact |
| Exposure to ethosuximide (%) | 0 (0) | 8 (27) | 0.302 | Fisher’s exact |
| Exposure to gabapentin (%) | 1 (17) | 9 (30) | 0.655 | Fisher’s exact |
| Exposure to lacosamide (%) | 0 (0) | 4 (13) | 1 | Fisher’s exact |
| Exposure to levetiracetam (%) | 3 (50) | 23 (77) | 0.317 | Fisher’s exact |
| Exposure to lamotrigine (%) | 5 (83) | 26 (87) | 1 | Fisher’s exact |
| Exposure to oxcarbazepine (%) | 1 (17) | 3 (10) | 0.535 | Fisher’s exact |
| Exposure to phenobarbitone (%) | 4 (67) | 21 (70) | 1 | Fisher’s exact |
| Exposure to phenytoin (%) | 2 (33) | 22 (73) | 0.149 | Fisher’s exact |
| Exposure to pregabalin (%) | 0 (0) | 2 (7) | 1 | Fisher’s exact |
| Exposure to primidone (%) | 0 (0) | 9 (30) | 0.303 | Fisher’s exact |
| Exposure to stiripentol (%) | 4 (67) | 8 (27) | 0.149 | Fisher’s exact |
| Exposure to topiramate (%) | 5 (83) | 20 (67) | 0.643 | Fisher’s exact |
| Exposure to vigabatrin (%) | 2 (33) | 14 (47) | 0.672 | Fisher’s exact |
| Exposure to sodium valproate (%) | 6 (100) | 29 (97) | 1 | Fisher’s exact |
| Exposure to zonisamide (%) | 0 (0) | 7 (23) | 0.317 | Fisher’s exact |
| Subject living alone in the 12-month period before last follow-up/death, n (%) | 0 (0) | 0 (0) | Not applicable | Not applicable |
| Convulsive seizures in the 12-month period before last follow-up/death, n (%)* | 6 (100) | 22 (82) | 0.556 | Fisher’s exact |
| History of nocturnal seizures in the 12-month period before last follow-up/death, n (%)* | 2 (33) | 19 (70) | 0.159 | Fisher’s exact |

Abbreviations: SUDEP = sudden unexpected death in epilepsy, SD = standard deviation, IQR = interquartile range, *n* = number.

***** Missing data**:** convulsive seizures in the 12-month period before last follow-up/death (*n* = 3); history of nocturnal seizures in the 12-month period before last follow-up/death (*n* = 3)

**Supplementary Table 3. *SCN1A* mutations identified prior to WES in the Dravet Syndrome patients who died of SUDEP.**

| **ID** | **Variant Type** | **cDNA position** | **Predicted protein change** | **Inheritance** | **Number of mutations** | **SUDEP classification** |
| --- | --- | --- | --- | --- | --- | --- |
| 1 | splice site | c.4339-14T>G | unknown | de novo | 1 | Definite |
| 2 | nonsense | c.1738C>T | p.Arg580Ter | de novo | 1 | Probable |
| 3 | frameshift | c.5536_5539delAAAC | p.Lys1846SerfsTer11 | de novo | 1 | Probable |
| 4 | nonsense | c.1837C>T | p.Arg613Ter | de novo | 1 | Definite |
| 5 | frameshift | c.5536_5539delAAAC | p.Lys1846SerfsX11 | de novo | 1 | Definite |
| 6 | missense | c.4181C>T | p.Thr1394lle | de novo | 1 | Definite |

Abbreviations: WES = whole exome sequencing, SUDEP = sudden unexpected death in epilepsy, ID = identification number, cDNA = complementary DNA.

**Supplementary Table 4. *SCN1A* mutations identified prior to WES in the living Dravet Syndrome cohort.**

| **ID** | **Type** | **cDNA position** | **Predicted protein change** | **Inheritance** | **Number of mutations** |
| --- | --- | --- | --- | --- | --- |
| 7 | frameshift | c.1714_1718delACAAG | p.Thr572ProfsTer5 | de novo | 1 |
| 8 | in-frame deletion | c.2725_2727delATG | p.Met909del | not determined | 1 |
| 9 | missence | c.2729A>C | p.Glu910Pro | de novo | 1 |
| 10 | missense | c.3797A>C | p.Glu1266Ala | de novo | 1 |
| 11 | missense | c.4384T>C | p.Tyr1462His | de novo | 1 |
| 12 | missense | c.4568T>C | p.lle1523Thr | de novo | 1 |
| 13 | splice site | c.264+4_264+7delAGTG | unknown | de novo | 1 |
| 14 | missense | c.5639G>A | p.Gly1880Glu | One parent analysed, mother negative | 1 |
| 15 | nonsense | c.992delT | p.Leu331Ter | de novo | 1 |
| 16 | missense | c.2792G>A | p.Arg931His | not determined | 1 |
| 17 | premature stop codon | c.4369_4372dupCTGT | p.Tyr1458SerfsTer29 | de novo | 1 |
| 18 | frameshift | c.111delC | p.Lys38AsnfsTer54 | de novo | 1 |
| 19 | nonsense | c.1152G>A | p.Trp384Ter | father deceased, mother negative | 1 |
| 20 | missense | c.512T>A | p.Ile171Lys | de novo | 1 |
| 21 | frameshift | c.4062delT | p.Ile1356TyrfsTer4 | de novo | 1 |
| 22 | frameshift | c.1209delT | p.Phe403LeufsTer12 | father deceased, mother negative | 1 |
| 23 | nonsense | c.664C>T | p.Arg222Ter | de novo | 1 |
| 24 | missense | c.2792G>A | p.Arg931His | de novo | 1 |
| 25 | missense | c.302G>A | p.Arg101Gln | father deceased, mother negative | 1 |
| 26 | frameshift | c.4949dupT | p.Lys1651GlnfsTer22 | de novo | 1 |
| 27 | missense | c.5119T>G | p.Phe1707Val | One parent analysed, mother negative | 1 |
| 28 | missense | c.2831T>A | p.Val944Glu | de novo | 1 |
| 29 | nonsense | c.4933C>T | p.Arg1645Ter | de novo | 1 |
| 30 | missense; nonsense | c.1811G>A; c.4573C>T | p.Arg604His; p.Arg1525Ter | father deceased, mother negative | 2 |
| 31 | nonsense | c.5436G>A | p.Trp1812Ter | de novo | 1 |
| 32 | splice site | c.2589+3A>T | unknown | de novo | 1 |
| 33 | Mutation not detected | | | | |
| 34 | Mutation not detected | | | | |
| 35 | Mutation not detected | | | | |
| 36 | Mutation not detected | | | | |

Abbreviations: WES = whole-exome sequencing, ID = identification number, cDNA = complementary DNA.

**Supplementary Table 5. Coefficients of variation of the cumulative per-individual burden scores after applying different methods for batch correction.**

The selected batch correction method with the lowest coefficient of variation in all samples (SUDEP, *n* = 18; epilepsy controls, *n* = 87; disease controls, *n* = 1,479) is shown in bold.

| **Batch correction method** | **Observations** | **Mean** | **Standard deviation** | **Coefficient of variation** |
| --- | --- | --- | --- | --- |
| Per variant |  |  |  |  |
| Sequencing rate ≥ 70% | 1,584 | 324.81 | 74.06 | 22.80 |
| **Sequencing rate ≥ 80%** | **1,584** | **271.44** | **61.15** | **22.53** |
| Sequencing rate ≥ 90% | 1,584 | 165.98 | 38.81 | 23.38 |
| Per target interval |  |  |  |  |
| Mean average coverage ≥ 10 | 1,584 | 444.92 | 101.27 | 22.76 |
| Mean average coverage ≥ 30 | 1,584 | 350.56 | 82.78 | 23.61 |
| Mean average coverage ≥ 50 | 1,584 | 180.57 | 45.58 | 25.24 |


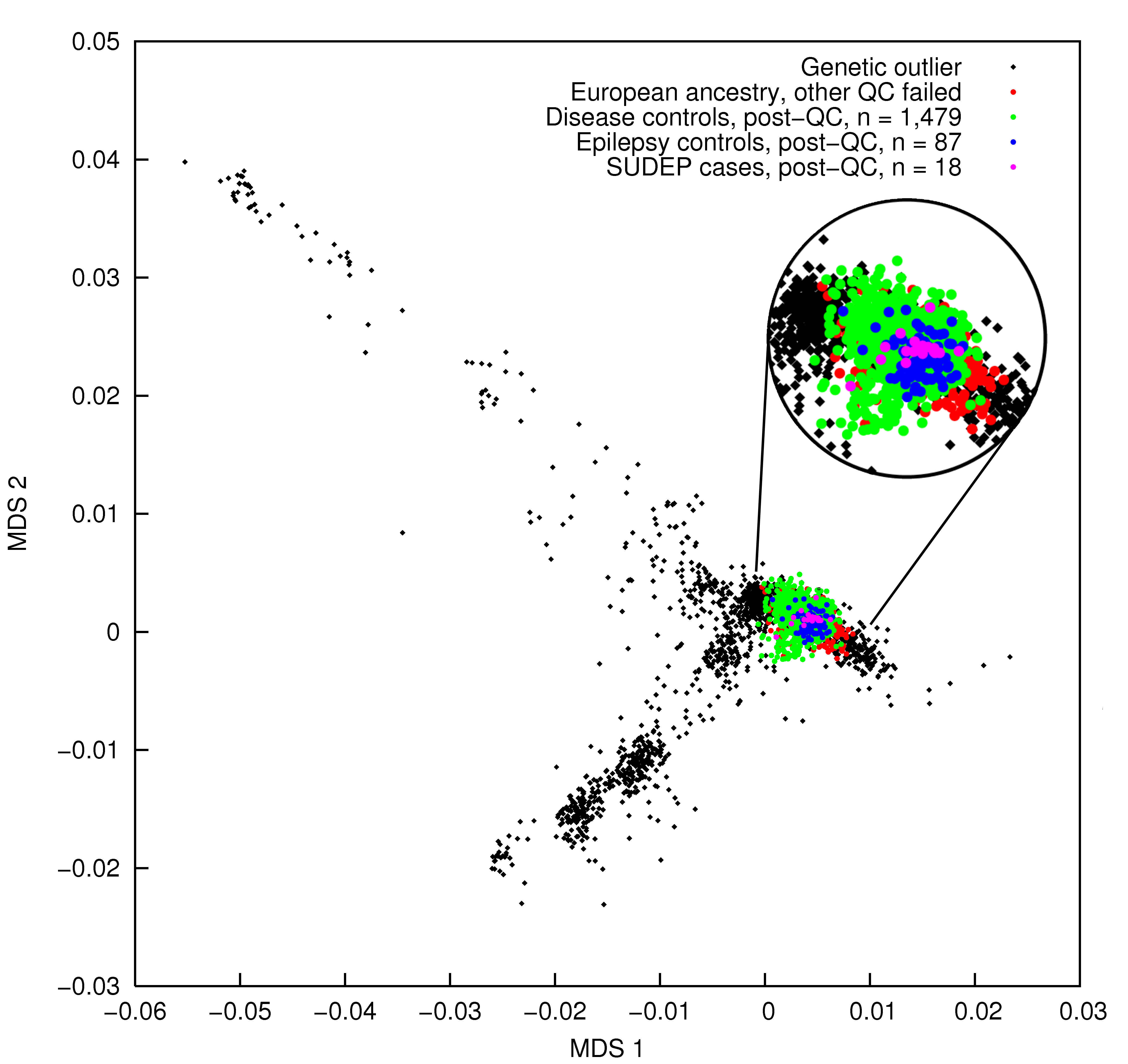


**Supplementary Figure 1. Multidimensional scaling analysis.** Plotted are 3,344 UCL-exomes samples after the first individual-level QC step (68 samples with low call rate filtered out). Each point within the scatter plot represents the individual coordinates of the first two dimensions of MDS analysis using 43,710 high-quality and uncorrelated variants. Genetic outliers for 20 MDS dimensions, indicated by black symbols, were removed as non-European samples from subsequent analyses. The zoomed area of the European cluster is indicated by black lines.


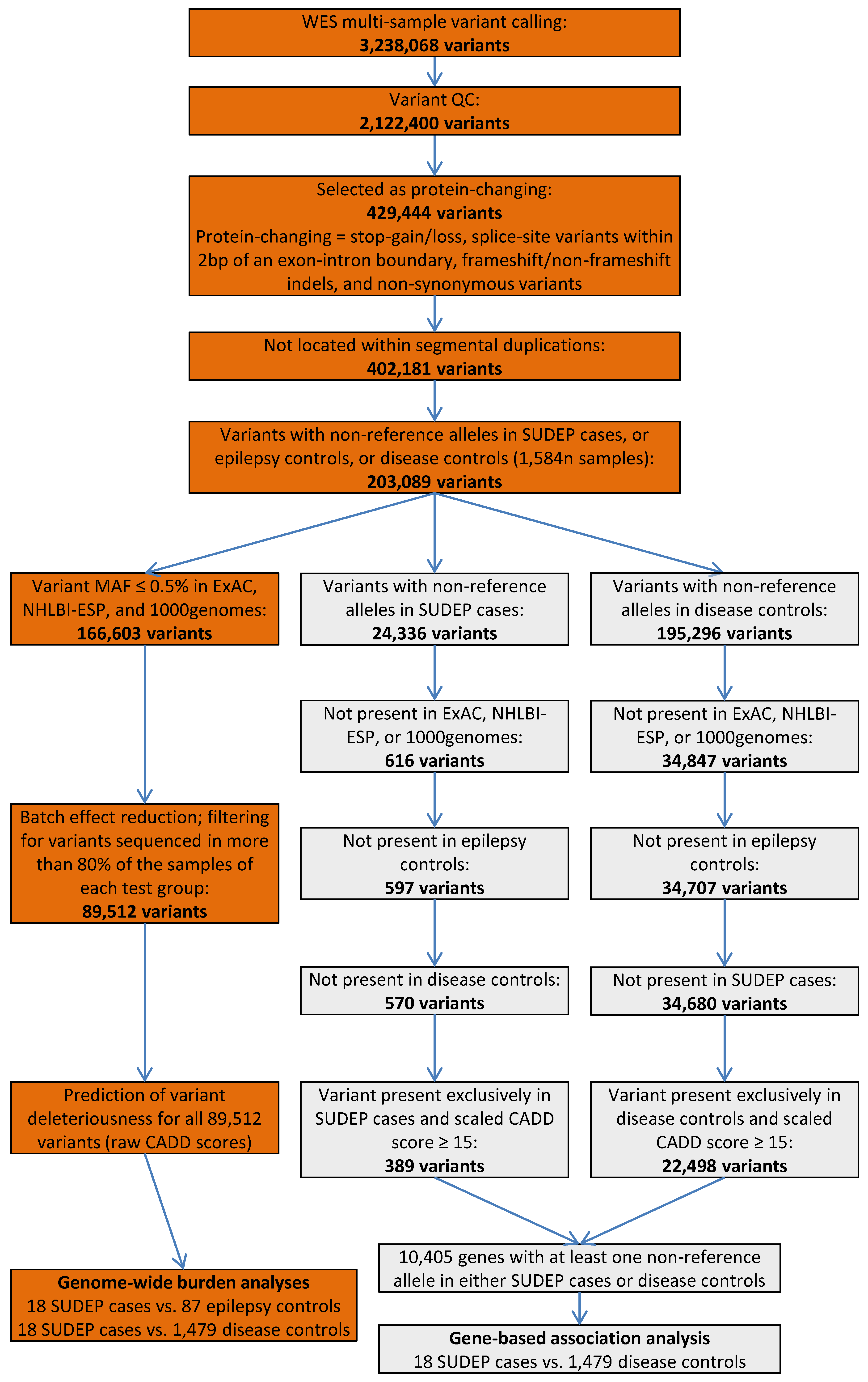


**Supplementary Figure 2. Study design and variant filtering flowchart.** Main part of the study (genome-wide burden analyses) is highlighted in dark orange. Secondary part (gene-based burden analyses) is highlighted in light grey. Abbreviations: WES = whole-exome sequencing, SUDEP = sudden unexpected death in epilepsy, ExAC = Exome Aggregation Consortium v0.2 non-Finnish Europeans (*n* = 34,427), NHLBI-ESP = NHLBI Grand Opportunity Exome Sequencing Project European-Americans (*n* = 4,300), 1000genomes = 1000 Genomes Phase 3 October 2014 Europeans (*n* = 503), MAF = minor allele frequency.


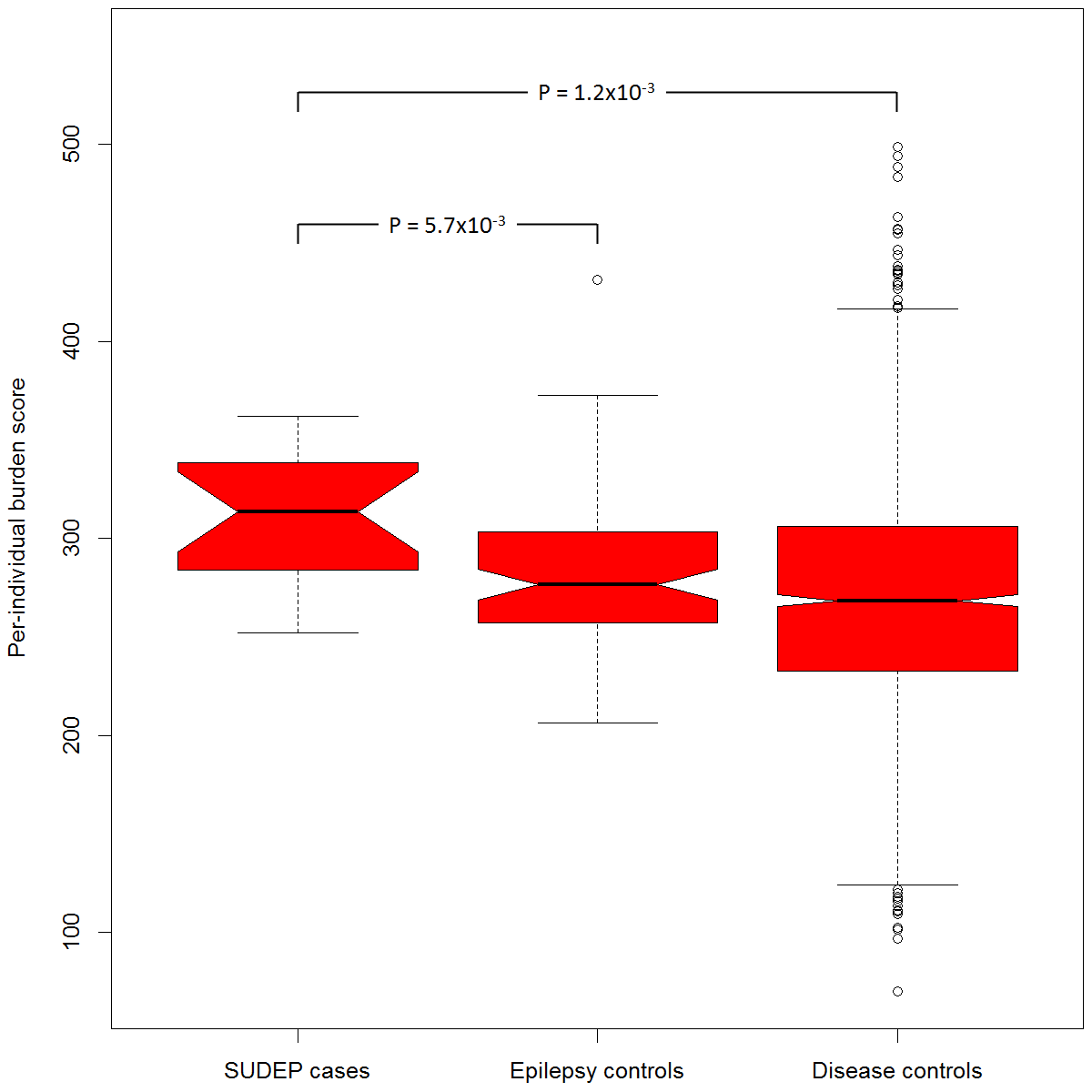


**Supplementary Figure 3. Notched boxplot of the per-individual burden scores.** Plotted are the per-individual burden scores for each test group. The thick black horizontal line is the median. The notched section represents the confidence interval around the median (median +/- 1.57 x IQR/n^0.5^). According to Chambers *et al.* (1983) (Graphical Methods for Data Analysis, p. 62), there is “strong evidence” (95% confidence) that their medians differ when the notches of two boxes do not overlap. The box represents the IQR, while the whiskers extend to the furthest observations within ± 1.5 IQR of the lower (first) quartile and the upper (third) quartile. Empty dots represent outliers beyond 1.5 IQRs.


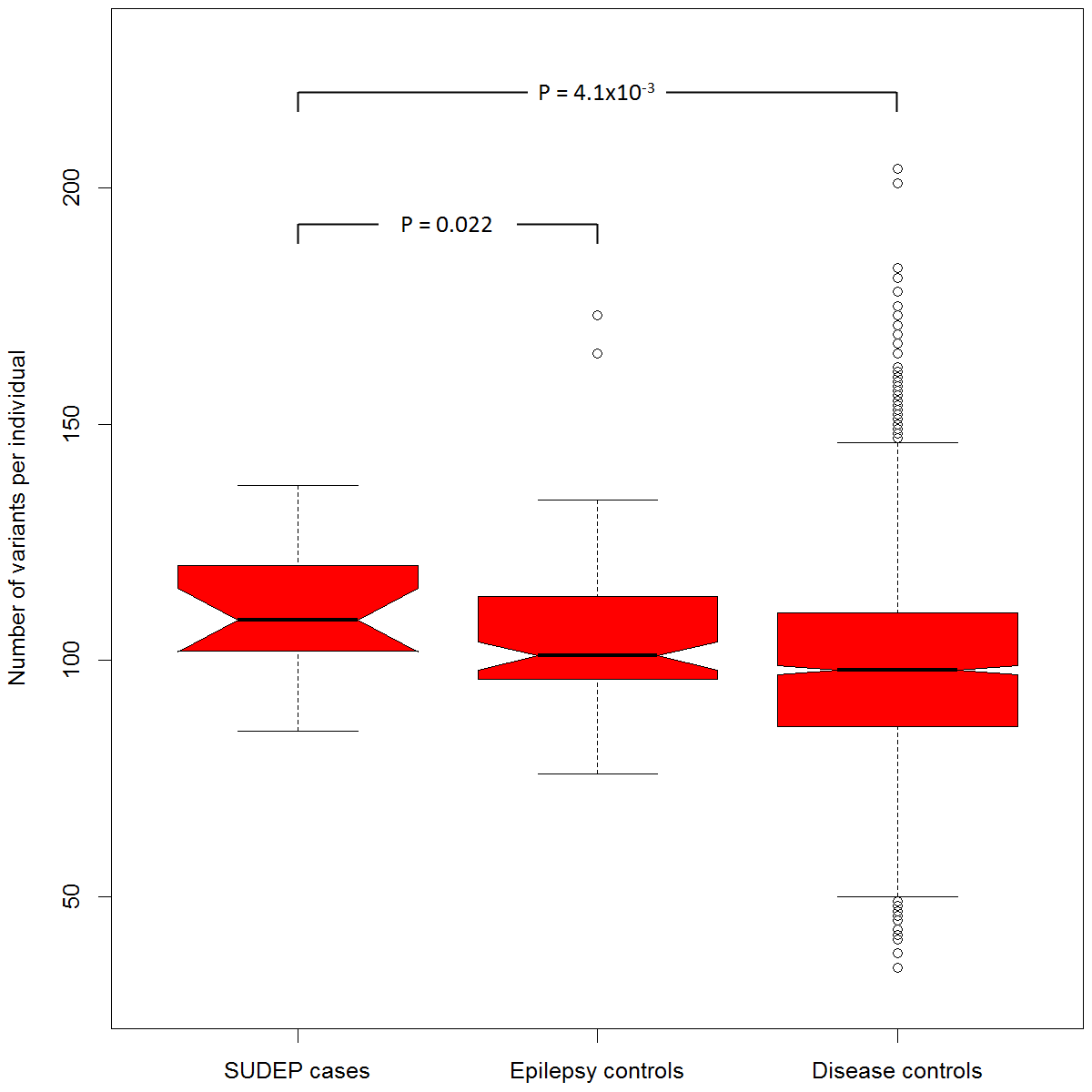


**Supplementary Figure 4. Notched boxplot of the number of variants per individual.** Plotted are the numbers of variants per individual of each test group. The thick black horizontal line is the median. The notched section represents the confidence interval around the median (median +/- 1.57 x IQR/n^0.5^). According to Chambers *et al.* (1983) (Graphical Methods for Data Analysis, p. 62), there is “strong evidence” (95% confidence) that their medians differ when the notches of two boxes do not overlap. The box represents the IQR, while the whiskers extend to the furthest observations within ± 1.5 IQR of the lower (first) quartile and the upper (third) quartile. Empty dots represent outliers beyond 1.5 IQRs.

**Supplementary Result 10: Whole exome sequencing coverage**

Coverage information was generated using the DepthOfCoverage module in GATK. The union of the Agilent, NimbleGen, and Illumina target regions was used in order to obtain uniform coverage statistics across all samples corresponding to the multi-sample call.

SUDEP samples: The mean average coverage across the union of all target intervals was 55x. On average, 56% of all target bases achieved 20x or greater coverage (range 47-60%). The mean average coverage across all hg19 Reference Sequence exons was 68x.

Epilepsy samples: The mean average coverage across the union of all target intervals was 40x. On average, 48% of all target bases achieved 20x or greater coverage (range 33-81%). The mean average coverage across all hg19 Reference Sequence exons was 50x.

UCL-exomes (disease control) samples: The mean average coverage across the union of all target intervals was 45x. On average, 51% of all target bases achieved 20x or greater coverage (range 16-88%). The mean average coverage across all hg19 Reference Sequence exons was 56x.

**Supplementary Result 11: Deleterious singleton variants in genes implicated in cardiac death or epilepsy causation**

Of 373 genes with at least one non-reference variant present in the SUDEP cohort only (Supplementary Table 7), we also found deleterious variants in one gene implicated in sudden cardiac death (*CACNB2* (Antzelevitch *et al.*, 2007)) and five genes implicated in different epilepsy syndromes (*CNTN2* (Stogmann *et al.*, 2013), *GABRG2* (Baulac *et al.*, 2001), *MAGI2* (Marshall *et al.*, 2008), *POLG* (Uusimaa *et al.*, 2013), and *SYNGAP1* (Carvill *et al.*, 2013)), each present as a singleton in the SUDEP cohort.

**Supplementary Table 6. Burden scores and variant numbers for the SUDEP and epilepsy control samples.**

The burden scores are calculated by summing the CADD scores for deleteriousness of every selected variant carried per individual**.**

| **ID** | **Group** | **Overall burden score** | **Number of variants** |
| --- | --- | --- | --- |
| 1 | SUDEP | 359.717 | 137 |
| 2 | SUDEP | 252.147 | 92 |
| 3 | SUDEP | 361.877 | 128 |
| 4 | SUDEP | 325.648 | 107 |
| 5 | SUDEP | 267.083 | 98 |
| 6 | SUDEP | 340.553 | 108 |
| 37 | SUDEP | 284.007 | 101 |
| 38 | SUDEP | 338.335 | 120 |
| 39 | SUDEP | 298.502 | 110 |
| 40 | SUDEP | 259.949 | 102 |
| 41 | SUDEP | 326.775 | 109 |
| 42 | SUDEP | 297.342 | 121 |
| 43 | SUDEP | 345.866 | 119 |
| 44 | SUDEP | 295.796 | 107 |
| 45 | SUDEP | 309.682 | 108 |
| 46 | SUDEP | 329.256 | 112 |
| 47 | SUDEP | 256.889 | 85 |
| 48 | SUDEP | 316.987 | 120 |
| 7 | Epilepsy control | 252.542 | 100 |
| 8 | Epilepsy control | 302.804 | 108 |
| 9 | Epilepsy control | 330.097 | 112 |
| 10 | Epilepsy control | 333.787 | 125 |
| 11 | Epilepsy control | 323.071 | 118 |
| 12 | Epilepsy control | 271.17 | 118 |
| 13 | Epilepsy control | 372.639 | 173 |
| 14 | Epilepsy control | 354.713 | 120 |
| 15 | Epilepsy control | 288.767 | 98 |
| 16 | Epilepsy control | 291.89 | 104 |
| 17 | Epilepsy control | 260.218 | 101 |
| 18 | Epilepsy control | 255.89 | 94 |
| 19 | Epilepsy control | 249.289 | 100 |
| 20 | Epilepsy control | 326.59 | 117 |
| 21 | Epilepsy control | 290.896 | 100 |
| 22 | Epilepsy control | 323.711 | 115 |
| 23 | Epilepsy control | 262.273 | 102 |
| 24 | Epilepsy control | 347.718 | 117 |
| 25 | Epilepsy control | 299.316 | 99 |
| 26 | Epilepsy control | 289.297 | 98 |
| 27 | Epilepsy control | 338.482 | 115 |
| 28 | Epilepsy control | 234.981 | 100 |
| 29 | Epilepsy control | 302.517 | 119 |
| 30 | Epilepsy control | 210.459 | 76 |
| 31 | Epilepsy control | 304.135 | 99 |
| 32 | Epilepsy control | 265.14 | 114 |
| 33 | Epilepsy control | 240.545 | 94 |
| 34 | Epilepsy control | 431.057 | 165 |
| 35 | Epilepsy control | 310.828 | 98 |
| 36 | Epilepsy control | 306.997 | 120 |
| 49 | Epilepsy control | 284.447 | 90 |
| 50 | Epilepsy control | 273.455 | 103 |
| 51 | Epilepsy control | 244.968 | 96 |
| 52 | Epilepsy control | 238.671 | 107 |
| 53 | Epilepsy control | 328.091 | 114 |
| 54 | Epilepsy control | 323.615 | 114 |
| 55 | Epilepsy control | 317.573 | 118 |
| 56 | Epilepsy control | 256.95 | 99 |
| 57 | Epilepsy control | 258.884 | 109 |
| 58 | Epilepsy control | 206.33 | 82 |
| 59 | Epilepsy control | 286.28 | 104 |
| 60 | Epilepsy control | 315.867 | 125 |
| 61 | Epilepsy control | 255.99 | 96 |
| 62 | Epilepsy control | 247.434 | 89 |
| 63 | Epilepsy control | 269.123 | 99 |
| 64 | Epilepsy control | 257.397 | 96 |
| 65 | Epilepsy control | 257.052 | 96 |
| 66 | Epilepsy control | 269.042 | 96 |
| 67 | Epilepsy control | 230.292 | 88 |
| 68 | Epilepsy control | 273.905 | 101 |
| 69 | Epilepsy control | 253.521 | 90 |
| 70 | Epilepsy control | 282.128 | 105 |
| 71 | Epilepsy control | 232.949 | 99 |
| 72 | Epilepsy control | 289.872 | 101 |
| 73 | Epilepsy control | 260.866 | 82 |
| 74 | Epilepsy control | 272.389 | 96 |
| 75 | Epilepsy control | 240.764 | 86 |
| 76 | Epilepsy control | 285.66 | 101 |
| 77 | Epilepsy control | 262.026 | 101 |
| 78 | Epilepsy control | 250.621 | 98 |
| 79 | Epilepsy control | 266.575 | 86 |
| 80 | Epilepsy control | 242.588 | 93 |
| 81 | Epilepsy control | 218.303 | 85 |
| 82 | Epilepsy control | 265.309 | 93 |
| 83 | Epilepsy control | 276.328 | 94 |
| 84 | Epilepsy control | 268.619 | 100 |
| 85 | Epilepsy control | 333.307 | 116 |
| 86 | Epilepsy control | 278.548 | 109 |
| 87 | Epilepsy control | 296.325 | 113 |
| 88 | Epilepsy control | 278.304 | 116 |
| 89 | Epilepsy control | 293.481 | 106 |
| 90 | Epilepsy control | 282.278 | 104 |
| 91 | Epilepsy control | 292.481 | 114 |
| 92 | Epilepsy control | 302.617 | 104 |
| 93 | Epilepsy control | 298.335 | 94 |
| 94 | Epilepsy control | 291.115 | 98 |
| 95 | Epilepsy control | 261.284 | 99 |
| 96 | Epilepsy control | 244.311 | 88 |
| 97 | Epilepsy control | 254.5 | 87 |
| 98 | Epilepsy control | 272.821 | 103 |
| 99 | Epilepsy control | 332.406 | 113 |
| 100 | Epilepsy control | 274.721 | 114 |
| 101 | Epilepsy control | 244.375 | 90 |
| 102 | Epilepsy control | 332.135 | 134 |
| 103 | Epilepsy control | 259.937 | 91 |
| 104 | Epilepsy control | 314.285 | 105 |
| 105 | Epilepsy control | 319.711 | 106 |

**Supplementary Table 7. List of all 373 genes with at least one non-reference variant in the SUDEP cases.**

Genes with *P*-values surpassing the Bonferroni-corrected threshold for significance (α = 1.56 x 10^-3^) are highlighted in grey. One gene with significant *P*-values but without Sanger confirmation is shown in red.

|  | Carrier of deleterious alleles | |  |  |  |
| --- | --- | --- | --- | --- | --- |
| Gene | Exclusive to SUDEP cases (*n*) | Exclusive to disease controls (*n*) | Burden | C-alpha | Comment |
|  |  |  | *P*-value | *P*-value |  |
| *SCN1A* | 2 | 4 | 1.21E-03 | 1.61E-04 | variants in SUDEP cases confirmed by Sanger sequencing |
| *LGI1* | 2 | 2 | 3.12E-04 | 3.12E-04 | variants in SUDEP cases confirmed by Sanger sequencing |
| *PIK3C2A* | 2 | 1 | 3.12E-04 | 3.34E-04 | one variant not confirmed by Sanger sequencing |
| *SMC4* | 2 | 1 | 5.39E-04 | 5.39E-04 | variants in SUDEP cases confirmed by Sanger sequencing |
| *COL6A3* | 2 | 5 | 7.27E-04 | 7.27E-04 | variants in SUDEP cases confirmed by Sanger sequencing |
| *TIE1* | 2 | 4 | 1.48E-03 | 2.01E-03 | variants in SUDEP cases confirmed by Sanger sequencing |
| *MAGI2* | 1 | 3 | 8.93E-03 | 1.21E-02 | epilepsy gene; variant in SUDEP case confirmed by Sanger sequencing |
| *GABRG2* | 1 | 0 | 9.80E-03 | 1.40E-02 | epilepsy gene; variant in SUDEP case confirmed by Sanger sequencing |
| *CACNB2* | 1 | 1 | 2.14E-02 | 2.14E-02 | sudden cardiac death gene; variant in SUDEP case confirmed by Sanger sequencing |
| *CNTN2* | 1 | 6 | 2.67E-02 | 2.49E-02 | epilepsy gene; variant in SUDEP case confirmed by Sanger sequencing |
| *POLG* | 1 | 3 | 4.01E-02 | 3.12E-02 | epilepsy gene; variant in SUDEP case confirmed by Sanger sequencing |
| *SYNGAP1* | 1 | 2 | 4.71E-01 | 5.88E-01 | epilepsy gene; variant in SUDEP case confirmed by Sanger sequencing |
| *DNAH8* | 2 | 11 | 4.92E-03 | 3.62E-03 |  |
| *VPS13D* | 2 | 7 | 3.61E-03 | 5.15E-03 |  |
| *SYNE1* | 2 | 23 | 9.95E-03 | 6.64E-03 |  |
| *MRPS6* | 2 | 1 | 1.02E-02 | 8.96E-03 |  |
| *TENM3* | 2 | 9 | 7.33E-03 | 9.42E-03 |  |
| *DNAH17* | 2 | 12 | 4.78E-01 | 3.91E-01 |  |
| *ANO7* | 1 | 0 | 2.47E-03 | 2.03E-03 |  |
| *HSPB7* | 1 | 0 | 2.34E-03 | 2.34E-03 |  |
| *SLC9A3* | 1 | 0 | 3.12E-03 | 3.34E-03 |  |
| *FLT3LG* | 1 | 0 | 4.04E-03 | 3.54E-03 |  |
| *CENPP* | 1 | 1 | 3.17E-03 | 3.62E-03 |  |
| *N4BP3* | 1 | 2 | 3.69E-03 | 3.69E-03 |  |
| *CPE* | 1 | 0 | 3.95E-03 | 3.95E-03 |  |
| *OSBPL6* | 1 | 0 | 7.39E-03 | 4.31E-03 |  |
| *FCN1* | 1 | 2 | 1.22E-02 | 4.38E-03 |  |
| *TOP3A* | 1 | 3 | 6.32E-03 | 4.42E-03 |  |
| *POMGNT2* | 1 | 0 | 4.96E-03 | 4.63E-03 |  |
| *TTC17* | 1 | 0 | 5.66E-03 | 4.67E-03 |  |
| *PRAF2* | 1 | 0 | 5.45E-03 | 4.77E-03 |  |
| *SLITRK2* | 1 | 1 | 1.48E-02 | 5.19E-03 |  |
| *MPP7* | 1 | 0 | 7.69E-03 | 5.38E-03 |  |
| *CD96* | 1 | 0 | 6.94E-03 | 5.40E-03 |  |
| *RPL13A* | 1 | 0 | 7.23E-03 | 5.42E-03 |  |
| *CYYR1* | 1 | 0 | 5.93E-03 | 5.53E-03 |  |
| *FOPNL* | 1 | 0 | 4.86E-03 | 5.55E-03 |  |
| *PITX2* | 1 | 0 | 4.25E-03 | 5.77E-03 |  |
| *XPNPEP1* | 1 | 0 | 5.06E-03 | 5.78E-03 |  |
| *R3HCC1L* | 1 | 0 | 7.87E-03 | 5.80E-03 |  |
| *SFTPB* | 1 | 0 | 4.34E-03 | 5.88E-03 |  |
| *PM20D2* | 1 | 0 | 6.31E-03 | 5.89E-03 |  |
| *DHX32* | 1 | 0 | 5.57E-03 | 5.94E-03 |  |
| *KLK7* | 1 | 0 | 6.38E-03 | 5.96E-03 |  |
| *KIAA1549* | 1 | 0 | 8.13E-03 | 5.99E-03 |  |
| *CRISP3* | 1 | 0 | 6.10E-03 | 6.10E-03 |  |
| *CHMP2A* | 1 | 0 | 8.73E-03 | 6.11E-03 |  |
| *TMED5* | 1 | 0 | 7.28E-03 | 6.37E-03 |  |
| *ACTR10* | 1 | 0 | 8.70E-03 | 6.41E-03 |  |
| *MAP10* | 1 | 0 | 6.42E-03 | 6.42E-03 |  |
| *ZNF264* | 1 | 0 | 6.93E-03 | 6.47E-03 |  |
| *ERCC1* | 1 | 0 | 8.49E-03 | 6.61E-03 |  |
| *PKLR* | 1 | 0 | 8.51E-03 | 6.62E-03 |  |
| *ST6GAL1* | 1 | 0 | 6.22E-03 | 6.66E-03 |  |
| *PTGES2* | 1 | 0 | 8.63E-03 | 6.71E-03 |  |
| *OTOA* | 1 | 0 | 5.59E-03 | 6.79E-03 |  |
| *PSENEN* | 1 | 0 | 6.40E-03 | 6.86E-03 |  |
| *THBS4* | 1 | 0 | 5.65E-03 | 6.87E-03 |  |
| *SLC25A3* | 1 | 0 | 9.87E-03 | 6.91E-03 |  |
| *TIMM9* | 1 | 0 | 7.90E-03 | 6.91E-03 |  |
| *ZNF513* | 1 | 0 | 6.46E-03 | 6.92E-03 |  |
| *OR2AP1* | 1 | 0 | 7.96E-03 | 6.96E-03 |  |
| *C12orf10* | 1 | 0 | 6.53E-03 | 7.00E-03 |  |
| *DNAJA3* | 1 | 0 | 8.01E-03 | 7.01E-03 |  |
| *TERT* | 1 | 0 | 6.58E-03 | 7.05E-03 |  |
| *ZNF451* | 1 | 0 | 6.18E-03 | 7.06E-03 |  |
| *PDZD3* | 1 | 0 | 6.24E-03 | 7.13E-03 |  |
| *HIST1H2BB* | 1 | 0 | 8.16E-03 | 7.14E-03 |  |
| *TACC1* | 1 | 0 | 5.90E-03 | 7.16E-03 |  |
| *PRSS21* | 1 | 0 | 8.29E-03 | 7.25E-03 |  |
| *NSMAF* | 1 | 0 | 6.79E-03 | 7.27E-03 |  |
| *SYAP1* | 1 | 0 | 8.44E-03 | 7.39E-03 |  |
| *MON1A* | 1 | 0 | 5.77E-03 | 7.41E-03 |  |
| *DMXL2* | 1 | 1 | 7.02E-03 | 7.53E-03 |  |
| *BOLL* | 1 | 0 | 9.19E-03 | 7.57E-03 |  |
| *MEX3A* | 1 | 1 | 1.03E-02 | 7.59E-03 |  |
| *ACTA2* | 1 | 0 | 8.16E-03 | 7.61E-03 |  |
| *WHSC1L1* | 1 | 1 | 7.71E-03 | 7.71E-03 |  |
| *CCDC9* | 1 | 0 | 8.83E-03 | 7.73E-03 |  |
| *ALOX12B* | 1 | 0 | 4.93E-03 | 7.75E-03 |  |
| *NLK* | 1 | 1 | 8.86E-03 | 7.76E-03 |  |
| *SPNS1* | 1 | 0 | 6.80E-03 | 7.77E-03 |  |
| *PIGR* | 1 | 1 | 1.01E-02 | 7.88E-03 |  |
| *SPTLC3* | 1 | 1 | 1.14E-02 | 8.01E-03 |  |
| *ZCCHC9* | 1 | 0 | 8.07E-03 | 8.07E-03 |  |
| *CPT1A* | 1 | 1 | 7.55E-03 | 8.09E-03 |  |
| *MTAP* | 1 | 0 | 5.43E-03 | 8.15E-03 |  |
| *PTPN5* | 1 | 0 | 1.05E-02 | 8.18E-03 |  |
| *OR4B1* | 1 | 0 | 8.27E-03 | 8.27E-03 |  |
| *SCML4* | 1 | 0 | 9.12E-03 | 8.51E-03 |  |
| *TROVE2* | 1 | 0 | 9.74E-03 | 8.52E-03 |  |
| *DYRK4* | 1 | 0 | 7.15E-03 | 8.68E-03 |  |
| *SUCO* | 1 | 0 | 8.68E-03 | 8.68E-03 |  |
| *GRHL3* | 1 | 0 | 6.43E-03 | 8.73E-03 |  |
| *RBM12* | 1 | 0 | 7.30E-03 | 8.86E-03 |  |
| *DLK2* | 1 | 1 | 1.46E-02 | 8.90E-03 |  |
| *RPL32* | 1 | 0 | 7.79E-03 | 8.90E-03 |  |
| *DSCAML1* | 1 | 0 | 1.02E-02 | 8.97E-03 |  |
| *TBCEL* | 1 | 0 | 7.89E-03 | 9.01E-03 |  |
| *WNT2B* | 1 | 1 | 1.10E-02 | 9.03E-03 |  |
| *MFAP1* | 1 | 0 | 7.17E-03 | 9.22E-03 |  |
| *TECPR2* | 1 | 1 | 1.08E-02 | 9.43E-03 |  |
| *TMEM95* | 1 | 1 | 8.35E-03 | 9.54E-03 |  |
| *CCDC60* | 1 | 0 | 7.47E-03 | 9.60E-03 |  |
| *DACH2* | 1 | 0 | 1.03E-02 | 9.62E-03 |  |
| *CORO1C* | 1 | 0 | 7.11E-03 | 9.65E-03 |  |
| *NUP88* | 1 | 3 | 1.38E-02 | 1.01E-02 |  |
| *NYNRIN* | 1 | 2 | 8.91E-03 | 1.02E-02 |  |
| *ADAMTS12* | 1 | 5 | 1.32E-02 | 1.03E-02 |  |
| *SLC24A4* | 1 | 1 | 1.03E-02 | 1.03E-02 |  |
| *TANGO2* | 1 | 0 | 8.03E-03 | 1.03E-02 |  |
| *NUMA1* | 1 | 2 | 1.48E-02 | 1.09E-02 |  |
| *TCF7L2* | 1 | 0 | 8.62E-03 | 1.11E-02 |  |
| *NR1H3* | 1 | 1 | 1.27E-02 | 1.11E-02 |  |
| *TTC7A* | 1 | 1 | 9.77E-03 | 1.12E-02 |  |
| *TSHZ3* | 1 | 0 | 1.05E-02 | 1.12E-02 |  |
| *DENND6A* | 1 | 0 | 1.20E-02 | 1.12E-02 |  |
| *INCA1* | 1 | 1 | 1.05E-02 | 1.13E-02 |  |
| *SLC7A1* | 1 | 0 | 1.30E-02 | 1.13E-02 |  |
| *OLFML2B* | 1 | 1 | 1.06E-02 | 1.14E-02 |  |
| *SIPA1L2* | 1 | 1 | 1.73E-02 | 1.15E-02 |  |
| *IPO5* | 1 | 1 | 1.57E-02 | 1.15E-02 |  |
| *CTH* | 1 | 1 | 1.51E-02 | 1.18E-02 |  |
| *NCKAP5* | 1 | 0 | 1.10E-02 | 1.18E-02 |  |
| *NARF* | 1 | 2 | 1.65E-02 | 1.21E-02 |  |
| *OR6C76* | 1 | 1 | 1.13E-02 | 1.21E-02 |  |
| *GLB1L2* | 1 | 3 | 1.31E-02 | 1.22E-02 |  |
| *PHKA2* | 1 | 1 | 1.22E-02 | 1.22E-02 |  |
| *PRPH2* | 1 | 1 | 1.57E-02 | 1.22E-02 |  |
| *CNBD2* | 1 | 1 | 8.63E-03 | 1.23E-02 |  |
| *STRN* | 1 | 1 | 1.59E-02 | 1.24E-02 |  |
| *MRPL50* | 1 | 0 | 1.25E-02 | 1.25E-02 |  |
| *WDR12* | 1 | 1 | 1.04E-02 | 1.27E-02 |  |
| *CWH43* | 1 | 1 | 1.12E-02 | 1.28E-02 |  |
| *UNC13A* | 1 | 1 | 1.74E-02 | 1.28E-02 |  |
| *VSIG8* | 1 | 0 | 8.97E-03 | 1.28E-02 |  |
| *PRPF39* | 1 | 1 | 1.75E-02 | 1.29E-02 |  |
| *NUDT9* | 1 | 1 | 1.29E-02 | 1.29E-02 |  |
| *MASP2* | 1 | 3 | 1.21E-02 | 1.30E-02 |  |
| *CSRNP3* | 1 | 0 | 1.08E-02 | 1.31E-02 |  |
| *CHI3L2* | 1 | 0 | 1.34E-02 | 1.34E-02 |  |
| *PPP3R1* | 1 | 1 | 1.64E-02 | 1.35E-02 |  |
| *CEP97* | 1 | 1 | 1.05E-02 | 1.35E-02 |  |
| *MFN1* | 1 | 1 | 1.11E-02 | 1.35E-02 |  |
| *ZNF48* | 1 | 2 | 1.74E-02 | 1.35E-02 |  |
| *STRIP2* | 1 | 2 | 1.55E-02 | 1.36E-02 |  |
| *MTBP* | 1 | 1 | 1.45E-02 | 1.36E-02 |  |
| *ACOXL* | 1 | 1 | 1.20E-02 | 1.37E-02 |  |
| *KIF5C* | 1 | 1 | 1.29E-02 | 1.38E-02 |  |
| *FN3KRP* | 1 | 1 | 1.08E-02 | 1.39E-02 |  |
| *OR3A1* | 1 | 1 | 1.22E-02 | 1.39E-02 |  |
| *KCNH1* | 1 | 1 | 1.09E-02 | 1.40E-02 |  |
| *CTRL* | 1 | 1 | 1.83E-02 | 1.43E-02 |  |
| *TNRC6C* | 1 | 1 | 1.14E-02 | 1.46E-02 |  |
| *POLR3B* | 1 | 2 | 1.77E-02 | 1.46E-02 |  |
| *ASXL1* | 1 | 1 | 1.67E-02 | 1.46E-02 |  |
| *COG2* | 1 | 1 | 1.31E-02 | 1.50E-02 |  |
| *SLC30A6* | 1 | 1 | 1.61E-02 | 1.50E-02 |  |
| *CCDC33* | 1 | 1 | 1.11E-02 | 1.51E-02 |  |
| *PPP1R12A* | 1 | 0 | 1.51E-02 | 1.51E-02 |  |
| *PAPPA* | 1 | 1 | 1.62E-02 | 1.51E-02 |  |
| *CHODL* | 1 | 2 | 1.53E-02 | 1.53E-02 |  |
| *ERAP1* | 1 | 1 | 1.34E-02 | 1.53E-02 |  |
| *TXNDC16* | 1 | 1 | 1.08E-02 | 1.55E-02 |  |
| *HHAT* | 1 | 2 | 1.44E-02 | 1.55E-02 |  |
| *LGALS13* | 1 | 1 | 1.67E-02 | 1.56E-02 |  |
| *THAP1* | 1 | 1 | 1.56E-02 | 1.56E-02 |  |
| *SCN11A* | 1 | 2 | 1.83E-02 | 1.60E-02 |  |
| *NDST4* | 1 | 1 | 1.26E-02 | 1.62E-02 |  |
| *ENOX1* | 1 | 1 | 1.86E-02 | 1.63E-02 |  |
| *EBNA1BP2* | 1 | 1 | 1.87E-02 | 1.63E-02 |  |
| *KCNB2* | 1 | 3 | 1.53E-02 | 1.64E-02 |  |
| *FAM115A* | 1 | 1 | 1.76E-02 | 1.65E-02 |  |
| *OLFML3* | 1 | 3 | 3.65E-02 | 1.65E-02 |  |
| *CAD* | 1 | 7 | 1.49E-02 | 1.70E-02 |  |
| *BAI3* | 1 | 1 | 1.19E-02 | 1.70E-02 |  |
| *MRPS28* | 1 | 0 | 1.95E-02 | 1.71E-02 |  |
| *RBM28* | 1 | 1 | 1.72E-02 | 1.72E-02 |  |
| *EBF2* | 1 | 1 | 1.21E-02 | 1.72E-02 |  |
| *RBCK1* | 1 | 1 | 1.42E-02 | 1.73E-02 |  |
| *PZP* | 1 | 1 | 1.61E-02 | 1.73E-02 |  |
| *CCNL2* | 1 | 1 | 1.85E-02 | 1.73E-02 |  |
| *MDGA1* | 1 | 2 | 1.35E-02 | 1.73E-02 |  |
| *BUB1B* | 1 | 3 | 2.12E-02 | 1.75E-02 |  |
| *IL1RN* | 1 | 1 | 2.14E-02 | 1.76E-02 |  |
| *TAC1* | 1 | 1 | 2.14E-02 | 1.77E-02 |  |
| *TMED8* | 1 | 1 | 2.44E-02 | 1.79E-02 |  |
| *TACR1* | 1 | 1 | 2.07E-02 | 1.81E-02 |  |
| *BECN1* | 1 | 3 | 1.50E-02 | 1.82E-02 |  |
| *KLHDC7A* | 1 | 2 | 1.61E-02 | 1.84E-02 |  |
| *LRSAM1* | 1 | 2 | 1.85E-02 | 1.85E-02 |  |
| *FAM171A1* | 1 | 2 | 2.12E-02 | 1.86E-02 |  |
| *ZNF365* | 1 | 2 | 1.87E-02 | 1.87E-02 |  |
| *LCMT2* | 1 | 3 | 1.92E-02 | 1.92E-02 |  |
| *MAGEC2* | 1 | 1 | 2.89E-02 | 1.93E-02 |  |
| *GOLGA4* | 1 | 4 | 2.50E-02 | 1.94E-02 |  |
| *ART3* | 1 | 1 | 2.09E-02 | 1.96E-02 |  |
| *GPALPP1* | 1 | 1 | 1.96E-02 | 1.96E-02 |  |
| *DLC1* | 1 | 2 | 2.24E-02 | 1.96E-02 |  |
| *RAB35* | 1 | 1 | 1.97E-02 | 1.97E-02 |  |
| *CDK15* | 1 | 2 | 1.63E-02 | 1.98E-02 |  |
| *NOC3L* | 1 | 1 | 2.12E-02 | 1.98E-02 |  |
| *GLI2* | 1 | 3 | 2.83E-02 | 1.98E-02 |  |
| *SIL1* | 1 | 3 | 2.13E-02 | 1.99E-02 |  |
| *FAM168A* | 1 | 3 | 2.43E-02 | 2.00E-02 |  |
| *DGKB* | 1 | 2 | 2.00E-02 | 2.00E-02 |  |
| *NEMF* | 1 | 2 | 2.47E-02 | 2.03E-02 |  |
| *SLC17A5* | 1 | 2 | 2.34E-02 | 2.04E-02 |  |
| *CD97* | 1 | 1 | 1.69E-02 | 2.05E-02 |  |
| *GAS2L3* | 1 | 1 | 1.92E-02 | 2.05E-02 |  |
| *POLI* | 1 | 2 | 1.38E-02 | 2.07E-02 |  |
| *CTNS* | 1 | 2 | 1.41E-02 | 2.11E-02 |  |
| *MED12L* | 1 | 3 | 3.34E-02 | 2.12E-02 |  |
| *ILF3* | 1 | 3 | 1.99E-02 | 2.14E-02 |  |
| *HK2* | 1 | 2 | 2.60E-02 | 2.14E-02 |  |
| *TPBG* | 1 | 1 | 2.01E-02 | 2.15E-02 |  |
| *MAB21L1* | 1 | 1 | 2.62E-02 | 2.15E-02 |  |
| *ETFA* | 1 | 2 | 2.47E-02 | 2.16E-02 |  |
| *ZC3H7A* | 1 | 2 | 1.53E-02 | 2.19E-02 |  |
| *PID1* | 1 | 2 | 2.19E-02 | 2.19E-02 |  |
| *ZNF223* | 1 | 3 | 2.19E-02 | 2.19E-02 |  |
| *IMPG2* | 1 | 1 | 2.36E-02 | 2.20E-02 |  |
| *IPO9* | 1 | 1 | 2.06E-02 | 2.21E-02 |  |
| *ATXN2* | 1 | 4 | 1.56E-02 | 2.22E-02 |  |
| *MTMR3* | 1 | 1 | 2.54E-02 | 2.23E-02 |  |
| *CDH7* | 1 | 1 | 2.24E-02 | 2.24E-02 |  |
| *FRMPD3* | 1 | 4 | 1.31E-02 | 2.25E-02 |  |
| *PHGDH* | 1 | 2 | 2.11E-02 | 2.26E-02 |  |
| *NBEA* | 1 | 3 | 3.40E-02 | 2.27E-02 |  |
| *NUB1* | 1 | 1 | 1.49E-02 | 2.34E-02 |  |
| *LRRC17* | 1 | 2 | 2.51E-02 | 2.35E-02 |  |
| *GPATCH3* | 1 | 2 | 1.96E-02 | 2.38E-02 |  |
| *CCNB2* | 1 | 2 | 2.39E-02 | 2.39E-02 |  |
| *ATM* | 1 | 6 | 3.42E-02 | 2.40E-02 |  |
| *PRDM16* | 1 | 1 | 2.40E-02 | 2.40E-02 |  |
| *CNTNAP5* | 1 | 3 | 2.59E-02 | 2.41E-02 |  |
| *IL16* | 1 | 3 | 2.99E-02 | 2.46E-02 |  |
| *NCOA2* | 1 | 2 | 2.07E-02 | 2.51E-02 |  |
| *ATP9A* | 1 | 3 | 2.53E-02 | 2.53E-02 |  |
| *ZNF470* | 1 | 2 | 2.72E-02 | 2.54E-02 |  |
| *GNPDA1* | 1 | 2 | 2.76E-02 | 2.58E-02 |  |
| *NDE1* | 1 | 2 | 2.96E-02 | 2.59E-02 |  |
| *ERCC3* | 1 | 3 | 2.28E-02 | 2.61E-02 |  |
| *PKD1L1* | 1 | 3 | 2.46E-02 | 2.64E-02 |  |
| *RABGAP1L* | 1 | 4 | 3.21E-02 | 2.65E-02 |  |
| *FNIP1* | 1 | 3 | 1.96E-02 | 2.66E-02 |  |
| *CWF19L1* | 1 | 2 | 2.48E-02 | 2.66E-02 |  |
| *NCF2* | 1 | 3 | 2.86E-02 | 2.67E-02 |  |
| *TRAPPC9* | 1 | 2 | 1.97E-02 | 2.67E-02 |  |
| *AKT1* | 1 | 3 | 2.69E-02 | 2.69E-02 |  |
| *BRCA2* | 1 | 6 | 2.51E-02 | 2.69E-02 |  |
| *NFAT5* | 1 | 3 | 2.36E-02 | 2.69E-02 |  |
| *MLLT4* | 1 | 0 | 3.09E-02 | 2.71E-02 |  |
| *RBM23* | 1 | 3 | 2.74E-02 | 2.74E-02 |  |
| *NR1H4* | 1 | 2 | 3.23E-02 | 2.82E-02 |  |
| *CAPN11* | 1 | 1 | 2.00E-02 | 2.85E-02 |  |
| *FERMT1* | 1 | 4 | 2.51E-02 | 2.87E-02 |  |
| *TBC1D15* | 1 | 1 | 2.37E-02 | 2.88E-02 |  |
| *PNMAL1* | 1 | 3 | 2.03E-02 | 2.90E-02 |  |
| *CCKAR* | 1 | 4 | 3.11E-02 | 2.90E-02 |  |
| *CEP76* | 1 | 7 | 3.37E-02 | 2.95E-02 |  |
| *ZDHHC5* | 1 | 1 | 3.24E-02 | 3.02E-02 |  |
| *COL6A6* | 1 | 2 | 1.96E-02 | 3.08E-02 |  |
| *RFX2* | 1 | 3 | 2.54E-02 | 3.09E-02 |  |
| *ATXN7* | 1 | 3 | 4.95E-02 | 3.15E-02 |  |
| *LDHAL6A* | 1 | 1 | 2.95E-02 | 3.16E-02 |  |
| *PRCP* | 1 | 3 | 3.66E-02 | 3.20E-02 |  |
| *CD207* | 1 | 3 | 3.90E-02 | 3.21E-02 |  |
| *PARD3* | 1 | 3 | 3.45E-02 | 3.22E-02 |  |
| *ITGAE* | 1 | 3 | 2.38E-02 | 3.23E-02 |  |
| *PSD4* | 1 | 3 | 2.83E-02 | 3.23E-02 |  |
| *GPR37* | 1 | 3 | 4.10E-02 | 3.33E-02 |  |
| *SOX6* | 1 | 2 | 4.56E-02 | 3.36E-02 |  |
| *PRKCH* | 1 | 3 | 3.38E-02 | 3.38E-02 |  |
| *CFAP43* | 1 | 3 | 5.33E-02 | 3.39E-02 |  |
| *MTRF1* | 1 | 3 | 2.81E-02 | 3.41E-02 |  |
| *PSTK* | 1 | 2 | 3.95E-02 | 3.42E-02 |  |
| *PRKD3* | 1 | 4 | 2.31E-02 | 3.47E-02 |  |
| *SEC24C* | 1 | 5 | 3.48E-02 | 3.48E-02 |  |
| *DLD* | 1 | 2 | 3.49E-02 | 3.49E-02 |  |
| *VPS41* | 1 | 5 | 6.23E-02 | 3.52E-02 |  |
| *SALL4* | 1 | 3 | 3.33E-02 | 3.57E-02 |  |
| *CEP128* | 1 | 4 | 3.60E-02 | 3.60E-02 |  |
| *TFDP2* | 1 | 1 | 3.01E-02 | 3.66E-02 |  |
| *MSL2* | 1 | 3 | 3.26E-02 | 3.72E-02 |  |
| *TTC3* | 1 | 4 | 4.87E-02 | 3.72E-02 |  |
| *GUCY1A2* | 1 | 1 | 3.09E-02 | 3.75E-02 |  |
| *NUP98* | 1 | 3 | 3.54E-02 | 3.80E-02 |  |
| *ABHD8* | 1 | 1 | 3.53E-02 | 3.80E-02 |  |
| *ANKRD50* | 1 | 4 | 3.82E-02 | 3.82E-02 |  |
| *NBAS* | 1 | 4 | 4.19E-02 | 3.89E-02 |  |
| *MKI67* | 1 | 6 | 5.42E-02 | 3.92E-02 |  |
| *TRPM7* | 1 | 3 | 2.50E-02 | 3.92E-02 |  |
| *PSMD13* | 1 | 3 | 4.02E-02 | 4.02E-02 |  |
| *ARHGAP30* | 1 | 6 | 5.70E-02 | 4.11E-02 |  |
| *MARCH8* | 1 | 4 | 5.43E-02 | 4.15E-02 |  |
| *PIKFYVE* | 1 | 3 | 4.22E-02 | 4.22E-02 |  |
| *PCNXL4* | 1 | 1 | 4.05E-02 | 4.36E-02 |  |
| *PLCG2* | 1 | 4 | 2.83E-02 | 4.45E-02 |  |
| *TDRD9* | 1 | 6 | 9.31E-02 | 4.48E-02 |  |
| *NUP205* | 1 | 8 | 4.18E-02 | 4.50E-02 |  |
| *NFATC3* | 1 | 5 | 3.14E-02 | 4.71E-02 |  |
| *ATF7IP* | 1 | 4 | 4.17E-02 | 4.81E-02 |  |
| *CLUH* | 1 | 3 | 3.06E-02 | 4.81E-02 |  |
| *SLC25A32* | 1 | 2 | 4.92E-02 | 4.92E-02 |  |
| *ITPR1* | 1 | 7 | 5.08E-02 | 5.08E-02 |  |
| *GBA2* | 1 | 4 | 2.55E-02 | 5.10E-02 |  |
| *OR5M10* | 1 | 2 | 6.00E-02 | 5.20E-02 |  |
| *PIGN* | 1 | 3 | 4.00E-02 | 5.23E-02 |  |
| *NCAPD3* | 1 | 3 | 4.87E-02 | 5.24E-02 |  |
| *PHLDB2* | 1 | 4 | 6.15E-02 | 5.33E-02 |  |
| *KIAA2018* | 1 | 6 | 4.68E-02 | 5.40E-02 |  |
| *TTLL6* | 1 | 3 | 6.96E-02 | 5.65E-02 |  |
| *EML5* | 1 | 4 | 6.70E-02 | 5.80E-02 |  |
| *CYP2R1* | 1 | 5 | 8.52E-02 | 5.83E-02 |  |
| *CCDC141* | 1 | 7 | 5.88E-02 | 5.88E-02 |  |
| *PTPRT* | 1 | 3 | 3.83E-02 | 5.90E-02 |  |
| *USP24* | 1 | 8 | 1.03E-01 | 6.07E-02 |  |
| *MATN2* | 1 | 4 | 4.83E-02 | 6.32E-02 |  |
| *GPR125* | 1 | 5 | 6.47E-02 | 6.47E-02 |  |
| *LRP1B* | 1 | 14 | 9.60E-02 | 6.57E-02 |  |
| *THADA* | 1 | 8 | 1.12E-01 | 6.95E-02 |  |
| *KCNQ5* | 1 | 4 | 5.65E-02 | 6.96E-02 |  |
| *ERICH2* | 1 | 2 | 6.07E-02 | 7.01E-02 |  |
| *PCDH15* | 1 | 6 | 5.73E-02 | 7.05E-02 |  |
| *DNAH3* | 1 | 11 | 8.20E-02 | 7.10E-02 |  |
| *PTPRD* | 1 | 5 | 6.44E-02 | 7.43E-02 |  |
| *MYO10* | 1 | 6 | 5.49E-02 | 7.59E-02 |  |
| *PPL* | 1 | 38 | 7.30E-02 | 7.87E-02 |  |
| *LAMA2* | 1 | 8 | 8.54E-02 | 7.93E-02 |  |
| *FER1L6* | 1 | 7 | 9.76E-02 | 7.93E-02 |  |
| *FRYL* | 1 | 6 | 6.63E-02 | 8.67E-02 |  |
| *CDH1* | 1 | 6 | 7.18E-02 | 8.84E-02 |  |
| *LAMC1* | 1 | 8 | 7.56E-02 | 1.05E-01 |  |
| *PKHD1L1* | 1 | 14 | 2.37E-01 | 2.03E-01 |  |
| *PITPNM1* | 1 | 2 | 5.33E-01 | 3.33E-01 |  |
| *LOXHD1* | 1 | 14 | 5.00E-01 | 3.75E-01 |  |
| *BEAN1* | 1 | 2 | 5.22E-01 | 3.91E-01 |  |
| *TRIM56* | 1 | 1 | 6.09E-01 | 3.91E-01 |  |
| *MARK2* | 1 | 2 | 2.86E-01 | 4.29E-01 |  |
| *MKNK1* | 1 | 2 | 3.57E-01 | 4.29E-01 |  |
| *PI4KA* | 1 | 2 | 4.44E-01 | 4.44E-01 |  |
| *USP25* | 1 | 2 | 6.67E-01 | 4.44E-01 |  |
| *MPP3* | 1 | 1 | 6.11E-01 | 4.44E-01 |  |
| *ARHGEF1* | 1 | 2 | 4.29E-01 | 4.76E-01 |  |
| *CACNA1B* | 1 | 7 | 6.25E-01 | 5.00E-01 |  |
| *MICAL3* | 1 | 6 | 6.88E-01 | 5.00E-01 |  |
| *RARG* | 1 | 2 | 5.63E-01 | 5.00E-01 |  |
| *COL11A2* | 1 | 6 | 5.33E-01 | 5.33E-01 |  |
| *ASB6* | 1 | 4 | 5.33E-01 | 5.33E-01 |  |
| *EPHB2* | 1 | 3 | 5.33E-01 | 5.33E-01 |  |
| *GLTPD2* | 1 | 3 | 6.92E-01 | 5.38E-01 |  |
| *PPAPDC2* | 1 | 1 | 5.38E-01 | 5.38E-01 |  |
| *MYO7A* | 1 | 9 | 4.44E-01 | 5.56E-01 |  |
| *ZNF831* | 1 | 3 | 3.91E-01 | 5.65E-01 |  |
| *JUN* | 1 | 2 | 8.33E-01 | 5.83E-01 |  |
| *PHF2* | 1 | 3 | 8.00E-01 | 6.00E-01 |  |
| *SYNPO2L* | 1 | 3 | 7.00E-01 | 6.00E-01 |  |
| *GAREM* | 1 | 1 | 3.91E-01 | 6.09E-01 |  |
| *NCOR2* | 1 | 9 | 5.00E-01 | 6.25E-01 |  |
| *ZNF335* | 1 | 3 | 4.09E-01 | 6.36E-01 |  |
| *TLE2* | 1 | 6 | 5.33E-01 | 6.67E-01 |  |
| *LAMA5* | 1 | 4 | 6.67E-01 | 6.67E-01 |  |
| *EMILIN1* | 1 | 2 | 4.44E-01 | 6.67E-01 |  |
| *COL6A2* | 1 | 7 | 5.38E-01 | 6.92E-01 |  |
| *TTN* | 1 | 85 | 7.14E-01 | 7.14E-01 |  |
| *RNH1* | 1 | 2 | 7.14E-01 | 7.14E-01 |  |
| *BLOC1S5* | 1 | 1 | 8.57E-01 | 7.14E-01 |  |
| *FBRSL1* | 1 | 3 | 4.44E-01 | 7.78E-01 |  |
| *OTOG* | 1 | 24 | 8.33E-01 | 8.33E-01 |  |
| *MAST2* | 1 | 12 | 8.33E-01 | 8.33E-01 |  |
| *STRA6* | 1 | 1 | 8.33E-01 | 8.33E-01 |  |
| *ITGA4* | 1 | 7 | 7.14E-01 | 8.57E-01 |  |

**References**

Antzelevitch C, Pollevick GD, Cordeiro JM, Casis O, Sanguinetti MC, Aizawa Y, et al. Loss-of-function mutations in the cardiac calcium channel underlie a new clinical entity characterized by ST-segment elevation, short QT intervals, and sudden cardiac death. Circulation 2007; 115: 442–449.

Van der Auwera GA, Carneiro MO, Hartl C, Poplin R, Del Angel G, Levy-Moonshine A, et al. From FastQ data to high confidence variant calls: the Genome Analysis Toolkit best practices pipeline. Curr Protoc Bioinformatics 2013; 11: 11.10.1–11.10.33.

Bailey JA, Yavor AM, Massa HF, Trask BJ, Eichler EE. Segmental duplications: organization and impact within the current human genome project assembly. Genome Res. 2001; 11: 1005–1017.

Baulac S, Huberfeld G, Gourfinkel-An I, Mitropoulou G, Beranger A, Prud’homme JF, et al. First genetic evidence of GABA(A) receptor dysfunction in epilepsy: a mutation in the gamma2-subunit gene. Nat. Genet. 2001; 28: 46–48.

Berg AT, Berkovic SF, Brodie MJ, Buchhalter J, Cross JH, van Emde Boas W, et al. Revised terminology and concepts for organization of seizures and epilepsies: report of the ILAE Commission on Classification and Terminology, 2005-2009. Epilepsia 2010; 51: 676–685.

Carvill GL, Heavin SB, Yendle SC, McMahon JM, O’Roak BJ, Cook J, et al. Targeted resequencing in epileptic encephalopathies identifies de novo mutations in CHD2 and SYNGAP1. Nat. Genet. 2013; 45: 825–830.

Danecek P, Auton A, Abecasis G, Albers CA, Banks E, DePristo MA, et al. The variant call format and VCFtools. Bioinformatics 2011; 27: 2156–2158.

DePristo MA, Banks E, Poplin R, Garimella KV, Maguire JR, Hartl C, et al. A framework for variation discovery and genotyping using next-generation DNA sequencing data. Nat. Genet. 2011; 43: 491–498.

Hunt KA, Mistry V, Bockett NA, Ahmad T, Ban M, Barker JN, et al. Negligible impact of rare autoimmune-locus coding-region variants on missing heritability. Nature 2013; 498: 232–235.

Kircher M, Witten DM, Jain P, O’Roak BJ, Cooper GM, Shendure J. A general framework for estimating the relative pathogenicity of human genetic variants. Nat. Genet. 2014; 46: 310–315.

Luedtke A, Powers S, Petersen A, Sitarik A, Bekmetjev A, Tintle NL. Evaluating methods for the analysis of rare variants in sequence data. BMC Proc 2011; 5 Suppl 9: S119.

Marshall CR, Young EJ, Pani AM, Freckmann M-L, Lacassie Y, Howald C, et al. Infantile spasms is associated with deletion of the MAGI2 gene on chromosome 7q11.23-q21.11. Am. J. Hum. Genet. 2008; 83: 106–111.

McKenna A, Hanna M, Banks E, Sivachenko A, Cibulskis K, Kernytsky A, et al. The Genome Analysis Toolkit: a MapReduce framework for analyzing next-generation DNA sequencing data. Genome Res. 2010; 20: 1297–1303.

Purcell S, Neale B, Todd-Brown K, Thomas L, Ferreira MAR, Bender D, et al. PLINK: a tool set for whole-genome association and population-based linkage analyses. Am. J. Hum. Genet. 2007; 81: 559–575.

Robinson JT, Thorvaldsdóttir H, Winckler W, Guttman M, Lander ES, Getz G, et al. Integrative genomics viewer. Nat. Biotechnol. 2011; 29: 24–26.

Smithson WH, Colwell B, Hanna J. Sudden unexpected death in epilepsy: addressing the challenges. Curr Neurol Neurosci Rep 2014; 14: 502.

Stogmann E, Reinthaler E, Eltawil S, El Etribi MA, Hemeda M, El Nahhas N, et al. Autosomal recessive cortical myoclonic tremor and epilepsy: association with a mutation in the potassium channel associated gene CNTN2. Brain 2013; 136: 1155–1160.

Tennessen JA, Bigham AW, O’Connor TD, Fu W, Kenny EE, Gravel S, et al. Evolution and functional impact of rare coding variation from deep sequencing of human exomes. Science 2012; 337: 64–69.

Uusimaa J, Gowda V, McShane A, Smith C, Evans J, Shrier A, et al. Prospective study of POLG mutations presenting in children with intractable epilepsy: prevalence and clinical features. Epilepsia 2013; 54: 1002–1011.

Wang K, Li M, Hakonarson H. ANNOVAR: functional annotation of genetic variants from high-throughput sequencing data. Nucleic Acids Res. 2010; 38: e164.
